# IGFBP7+ subpopulation and IGFBP7 risk score in astrocytoma: insights from scRNA-Seq and bulk RNA-Seq

**DOI:** 10.3389/fimmu.2024.1434300

**Published:** 2024-09-30

**Authors:** Liang Zhao, Wenwen Shao, Zhikai Xiahou, Li Ren, Chaobo Liu, Yanbing Song, Hao Xu, Zhihan Wang, Jin Xing

**Affiliations:** ^1^ Department of Neurosurgery, Shanghai Pudong Hospital, Fudan University Pudong Medical Center, Pudong, Shanghai, China; ^2^ First Clinical Medical College, Shandong University of Traditional Chinese Medicine, Jinan, China; ^3^ China Institute of Sport and Health Science, Beijing Sport University, Beijing, China

**Keywords:** astrocytoma, scRNA-seq, bulk RNA-seq, C0 IGFBP7+ glioma cells, prognosis

## Abstract

**Background:**

Glioma is the predominant malignant brain tumor that lacks effective treatment options due to its shielding by the blood-brain barrier (BBB). Astrocytes play a role in the development of glioma, yet the diverse cellular composition of astrocytoma has not been thoroughly researched.

**Methods:**

We examined the internal diversity of seven distinct astrocytoma subgroups through single-cell RNA sequencing (scRNA-seq), pinpointed crucial subgroups using CytoTRACE, monocle2 pseudotime analysis, and slingshot pseudotime analysis, employed various techniques to identify critical subgroups, and delved into cellular communication analysis. Then, we combined the clinical information of GBM patients and used bulk RNA sequencing (bulk RNA-seq) to analyze the prognostic impact of the relevant molecules on GBM patients, and we performed *in vitro* experiments for validation.

**Results:**

The analysis of the current study revealed that C0 IGFBP7+ Glioma cells were a noteworthy subpopulation of astrocytoma, influencing the differentiation and progression of astrocytoma. A predictive model was developed to categorize patients into high- and low-scoring groups based on the IGFBP7 Risk Score (IGRS), with survival analysis revealing a poorer prognosis for the high-IGRS group. Analysis of immune cell infiltration, identification of genes with differential expression, various enrichment analyses, assessment of copy number variations, and evaluation of drug susceptibility were conducted, all of which highlighted their significant influence on the prognosis of astrocytoma.

**Conclusion:**

This research enhances comprehension of the diverse cell composition of astrocytoma, delves into the various factors impacting the prognosis of astrocytoma, and offers fresh perspectives on treating glioma.

## Introduction

Glioma is a tumor caused by glial cells or precursor cells (1). Gliomas are the predominant histological form of primary cancer in the central nervous system, including high-grade gliomas and low-grade gliomas (2, 3). As for the classification, WHO advocates dividing gliomas into I–IV grades (4). Glioblastoma multiforme (GBM) is the predominant malignant brain tumor, making up 60%–70% of malignant gliomas (2), and is classified as a highly invasive grade IV glioma (5). Glioblastoma, also known as malignant glioma, is the deadliest type of brain tumor, typically resulting in a median survival time of 15 months (6), glioblastoma is the most aggressive form of astrocytoma. Prior research has indicated that there are gender disparities in the occurrence of GBM in adults, with a higher prevalence among males (1).

Treating a brain tumor can be challenging due to the presence of the blood-brain barrier (BBB), which protects it (6). At present, surgery, radiotherapy, and chemotherapy are still the main treatment methods for glioma (4). GBM cannot be removed surgically because of its invasive nature and ability to infiltrate normal surrounding brain tissue (7). At present, the main drugs for GBM chemotherapy are temozolomide, or TMZ. TMZ slightly improved the survival rate of patients but caused many side effects (6). The GBM tumor has strong resistance to radiotherapy and cytotoxic chemotherapy (7). Hence, there is no superior remedy for GBM, necessitating a more profound comprehension of the illness and investigation into novel treatment approaches. Recent literature has indicated that the combination of temozolomide therapy and tumor-treating fields (TTFields) can enhance both progression-free survival and overall survival in patients with glioblastoma (8). TTFields represents a therapeutic modality that combats mitosis, although further investigation is needed to fully elucidate its experimental findings. Moreover, this treatment necessitates the utilization of a device, which entails head hair shaving and may impose an additional burden on patients. The adoption of a multimodal standard therapy still entails an inevitable recurrence rate, with a median survival exceedingly merely one year (9), so other therapeutic modalities still need to be explored.

Single-cell analysis has become an important tool for dissecting cellular heterogeneity (10, 11). This method has been extensively utilized for examining the internal diversity of different types of cancer, including non-small cell lung cancer (12), melanoma (13), cervical cancer (14), bladder cancer (15), prostate cancer (16) and clear cell renal cell carcinomas (ccRCCs) (17–20), among others. The characteristics and makeup of the tumor immune microenvironment (TIME) play a crucial role in the treatment and outlook of tumors. Research has shown that astrocytes play a role in the development of glioma, indicating that this relationship could be a potential focus for novel treatments (21). Research has extensively shown that astrocytes have the ability to control the attraction of tumor-associated macrophages (TAMs) to the tumor microenvironment (TME) through CCL2, leading to the progression of glioblastoma by encouraging a pro-tumor phenotype in TAMs (22). However, the tumor immune microenvironment of astrocytoma has not been fully explored

For this research, we utilized scRNA-seq to analyze single-cell data from GBM patients. We conducted dimensionality reduction clustering analysis on astrocyte subpopulations, followed by inferCNV analysis to identify astrocytoma. Our goal was to investigate the diverse heterogeneity of astrocytoma subpopulations and identify key subpopulations with the potential for high differentiation. Additionally, we explored the transcription factors associated with these subpopulations. Furthermore, a risk assessment model was developed, and the infiltration of immune cells in tumors was investigated along with clinical data from patients with glioma. Finally, we performed *in vitro* experimental validation. These studies could offer fresh insights for treating GBM.

## Materials and methods

### Get glioma data

The Glioma single-cell RNA-seq data utilized in this study were obtained from the NCBI Gene Expression Omnibus (GEO) database at https://www.ncbi.nlm.nih.gov/geo/. The identification code for logging in was GSE182109.

Data pertaining to bulk RNA-seq was acquired from the Cancer Genome Atlas (TCGA) website (https://portal.gdc.cancer.gov/), which included clinical details (age, gender, ethnicity) and somatic mutation information for glioma patients.

### Raw data processing

The raw single-cell RNA data was analyzed using the “Seurat” package (version 4.3.0) (23, 24). To enhance data quality, the “DoubletFinder” R package (version 2.0.3) (17, 25) was utilized for eliminating doublet cells based on genetic data, followed by applying the “PercentageFeatureSet” function to filter out low-quality cells. High-quality cells meeting the criteria of (1) having 300 < nFeature < 7,500 genes detected in a single cell, (2) having 500 < nCount < 100,000 total transcriptomic count in a single cell, and (3) having the number of recognized genes in a single cell < 100,000 were retained. A single cell contains between 500 and 6,000 identifiable genes. Less than 20% of genes in a single cell were actively expressed by mitochondria.

### Data clustering analysis with reduced dimensions

High-quality glioma cells were acquired and then normalized using the “NormalizeData”function, followed by the identification of the top 2000 variable genes using the “FindVariableFeatures” function. All genes were centered using “ScaleData” (26–29). To remove batch effects across various samples, the samples were processed and analyzed using the “harmony” R package (version 0.1.1) (14, 30).

The initial 30 primary components (PCs) were reduced in size with the “RunPCA” function, then the glioma cells were grouped and examined using “FindClusters” and “FindNeighbors” categorized based on the marker genes of cell subgroups mentioned in previous studies, and displayed through Uniform Manifold Approximation and Projection (UMAP) (31).

### Detect astrocytoma utilizing InferCNV

By utilizing InferCNV (https://github.com/broadinstitute/inferCNV/wiki) (13), we were able to assess the astrocytes within the glioma cell subset and identify the differences in copy number within this subset. Taking EC (epithelial cell) as a control, the astrocytes with high-level copy number variation (CNV) were defined as astrocytoma.

### Subgroup identification of astrocytoma

By clustering astrocytoma, we were able to identify various subgroups, revealing its internal heterogeneity. First of all, the top 2,000 highly mutated genes in astrocytoma were identified, then normalized, and the “harmony” R package was applied to reduce batch effects. Finally, the first 30 principal components (PC) were projected onto the two-dimensional map by using the UMAP map, and the different subsets of astrocytoma were marked according to the marker genes in previous literature (32, 33).

Furthermore, we investigated the origin of tissues and the cell cycle of various cell subgroups, computed staging scores like G2M.Score and S.Score, and compared the variations in G2M.Score, S.Score, nFeature, and nCount across different cell subgroups.

### Identification and enrichment analysis of differentially expressed genes in astrocytoma subtypes

DEGs were identified for each astrocytoma subpopulation by screening with the “FindAllMarkers” function, detecting genes in a minimum of 25% of the cells with a false discovery rate (FDR) of less than 0.05 and an absolute log fold change (| logFCfilter |) greater than 1.

The “clusterProfiler” R package (version 0.1.1) (34, 35) was utilized for the analysis and enhancement of particular marker genes, with access to the Gene Ontology-Biological Processes (GOBP) database provided at http://www.geneontology.org (36, 37). During GO enrichment analysis, genes with p-values bel ow 0.05 were deemed to be statistically significant. Enriched entries were subjected to Gene Set Enrichment Analysis (GSEA) using gene sets obtained from the database (c2.cp.kegg.v7.5.1.symbols.gmt). Pathways that were significantly enriched were chosen using a false discovery rate (FDR) less than 0.05.

### Trajectory analysis of astrocytoma

Stemness and developmental trajectories of astrocytoma subpopulations were comprehensively inferred using a variety of analytical methods, including CytoTRACE analysis, monocle2 analysis, and the Slingshot method.

CytoTRACE can re-establish the relative differentiation status of astrocytoma subpopulations based on gene expression profiles (38) and assess the stemness of different cellular subpopulations.

A proposed time-series analysis of astrocytoma subpopulations was performed using the Monocle R package (version 2.24.0). Monocle identified cellular alterations during astrocytoma differentiation as a means of inferring the developmental trajectory of the subpopulation.

Slingshot analysis (version 2.6.0) was used to detect and generate multiple differentiation trajectories for the astrocytoma subpopulation. The “getlineage” and “getCurves” functions were used to infer subpopulation differentiation trajectories and to assess changes in cell expression levels over time, respectively.

### SCENIC analysis

To investigate the transcription factors (TFs) in the main subgroup, we utilized the pySCENIC algorithm to build a gene regulatory network, assessed the transcription factors’ expression, and unveiled the general distribution of the main subgroup transcription factors.

### Cell communication analysis

Astrocytoma subpopulations were analyzed for cellular communication using the ‘CellChat’ R package (version 1.6.1) (39), to examine and interpret inter-cellular communication networks derived from scRNA-seq data. The analysis was performed by integrating gene expression data from cells to establish the probability of communication through interactions between gene expression and signaling pathways, ligand-receptors, and their cofactors, which provided insights into the coordinated roles of signaling pathways in different cell types.

### Construction of risk score and establishment of nomogram

Prognosis-related genes and corresponding risk scores for each sample were obtained through univariate COX risk regression analysis using the “survival” R package (40, 41), as well as Least Absolute Shrinkage and Selection Operator (LASSO) Cox regression analysis (42–44) and multivariate COX risk regression. The risk score calculation formula: $Risk\;score=\sum_{i}^{n}Xi\times Yi$ (x: coefficient, y: gene expression level). According to the median risk score, the samples were divided into a high-risk group and a low-risk group. The prognostic features of various risk score categories were assessed using Kaplan-Meier survival analysis and the “timeROC” R package (45–47).

We assessed the predictive precision of risk scores by merging patient clinical data with risk scores for multivariate COX risk regression analysis. We developed a nomogram model to predict 1-, 3-, and 5-year overall survival (OS) in glioma patients, visualized it using the “rms” R package, assessed the model’s accuracy with c-index and ROC curves (48), and explored the relationship between model genes, risk scores, and OS.

### Immune microenvironment analysis

In order to evaluate the correlation between risk characteristics and the immune microenvironment, we used a combination of the ESTIMATE, CIBERSORT, and Xcell algorithms to comprehensively evaluate the immune microenvironment of astrocytoma patients. Furthermore, the CIBERSORT algorithm (http://cibersort.stanford.edu/). was utilized to examine the distribution of 22 various immune cell types. We computed the ImmuneScore, StromalScore, ESTIMATEScore, and TumorPurity values, along with the TIDE (TumorImmune Dysfunction and Exclusion) scores. In addition, the relationship between model genes, risk score, and OS was explored to illustrate the important role of genes in immune-related functions.

### Examining and enhancing the analysis of genes with varying expression levels in groups with high and low scores

The “DESeq2” was utilized to identify differentially expressed genes (DEGs) in groups with high and low risk scores, followed by enrichment analyses using the “clusterProfiler” R package (version 4.6.2) (49) for GO, Kyoto Encyclopedia of Genes and Genomes (KEGG) (50), and GSEA enrichment analyses.

### Tumor mutation analysis

Glioma patient somatic mutation information was obtained from the TCGA database, and the Tumor Mutation Burden (TMB) was assessed in various scoring categories using the “maftools” R package (51), and the subjects were classified into high TMB and low TMB according to the median TMB. Participants were divided into high TMB and low TMB groups using the median TMB value, and survival differences were compared between the two groups using Kaplan-Meier curves. Pearson correlation coefficients were used to analyze the relationship between score and TMB. Furthermore, we analyzed the genetic variation in gene copies (CNV) of the modeled genes.

### Drug sensitivity analysis

In order to better align with the clinical use of the drugs, we evaluated the sensitivity of the different drugs. The “pRRophetic” package (version 0.5) (52) was utilized to determine the IC50 value for each sample and assess the responsiveness of the groups with high and low risk scores.

### Cell culture

The U87 MG and U251 MG cell lines were acquired from the American Type Culture Collection (ATCC). The two cell types were grown in DMEM medium with 10% fetal bovine serum and 1% streptomycin/penicillin (Gibco BRL, USA) at 37°C, 5% CO2, and 95% humidity as per usual conditions.

### Cell transfection

Two small interfering RNAs (siRNAs) (GenePharma, Suzhou, China) were used to achieve FOSL2 knockdown, followed by inoculating cells in 6-well plates at 50% density. Transfection was performed with a negative control group (si-NC) and FOSL2 knockdown (si-FOSL2-1 and si-FOSL2-2). The transfection was carried out following the specific instructions provided by Lipofectamine 3000RNAiMAX (Invitrogen, USA).

### Cell viability assay

The viability of U87 MG and U251 MG cells that were transfected was measured by utilizing the Cell Counting Kit-8 (CCK-8, A311-01, Vazyme). Cell suspensions were added to 96-well plates (5 × 10^3^ cells per well) and left to incubate for 2 hours. The absorbance was then recorded at 450 nm on days 1, 2, 3, and 4. Mean optical density (OD) values were recorded, and the corresponding line graphs were plotted.

### Quantitative real-time PCR

Cell lines were used to extract total RNA with TRIzol reagent (15596018, Thermo), followed by cDNA synthesis using PrimeScript™ RT Reagent Kit (R232-01, Vazyme). cDNA was isolated using the SYBR Green Real-Time PCR Kit from TaKaRa Biotechnology in Dalian, China, through real-time quantitative PCR (qRT-PCR). The primers and siRNAs utilized in this research are displayed in **Supplementary Table 1**.

### Transwell

Cells (corning, USA) were either coated with or without matrix glue (BD Biosciences, USA) in a 24-well plate chamber. The cell suspension was then placed in the upper chamber with Costar and serum medium, while serum culture medium was added to the lower chamber. Put the cells in a cell incubator for 48 hours. Following incubation, the cells were treated with 4% paraformaldehyde and then stained with 0.1% crystal violet (Solarbio, China) to assess migration and invasion.

### Plate-cloning experiment

Transfected cells were seeded in a 6-well plate at a density of 1×10^3^ cells per well and incubated for 14 days. Next, the cells were rinsed with PBS and then treated with 4% paraformaldehyde (PFA) for a duration of 15 minutes. Finally, the cells were stained with 0.1% crystal violet (Solarbio, China) for 20 minutes and quantified.

### Wound healing

After transfection, the cells were grown in 6-well dishes until they reached 95% confluence, then a 200-mL sterile pipette was used to wash away debris with PBS in a straight line through the cell layer. Next, the serum-free solution was exchanged to sustain cell culture, and images of the wounds at the identical spot at 0 hours and 48 hours were captured for assessing the breadth of the wounds.

### 5-Ethyl-2’-deoxyuridine proliferation assay

U87 MG and U251 MG cell lines that were transfected were plated in 6-well cell culture plates with 5×103 cells per well and left at room temperature for 24 hours. After that, a solution made by EdU was added to serum-free medium and incubated for 2 hours at 37°. Next, the cells were rinsed with PBS and then treated with 4% paraformaldehyde for a duration of 30 minutes. Afterward, the cells were exposed to glycine (2 mg/mL) and 0.5% Triton X-100 for 15 minutes, followed by incubation with 1 mL of 1× Apollo and 1 mL of 1× Hoechst 33342 for 30 minutes. The quantification of cell proliferation was ultimately determined using fluorescence microscopy.

### Statistical analysis

The analysis of all the research was conducted using R software (version 4.3.0) and Python software (version 4.2.0). The Wilcoxon test, Pearson correlation coefficients, etc. Statistical tests were employed to evaluate the importance of variances among the groups (*P<0.05, **P<0.01, ***P<0.001).

## Results

### Main cell types of glioma

To comprehend the tumor microenvironment of glioma, we collected glioma cells from 18 patients following quality control of 234,148 high-quality cells. According to the marker genes, these high-quality cells were divided into 13 main cell types: microglia(49030), Myeloidcells (50565), Oligodendrocytes (29536), Astrocytes (46377), T_NK (28697), Excitatory_neuronal_cells (10997), Proliferating_cells (11346), Fibroblasts (1978), EndothelialCells(ECs)(1820), Muller_glia_cells (1580), B_Plasma(1245), Inhibitory_Neuronal _ Cells (814), Pericytes (163), and drawn into a 2D scatter plot by using Uniform Manifold Approximation and Projection (UMAP) technology (**Supplementary Figure 1A**). Additionally, we examined the tissue categories, cellular phases, and seurat groupings of each cell category, presenting them through UMAP visualizations paired with pie graphs (**Supplementary Figures 1B–D**). Bubble plots (**Supplementary Figure 1E**) displayed the top 5 marker genes for 13 cell types and 3 tissue types.

### Subtype identification of astrocytoma

**Figure 1:**
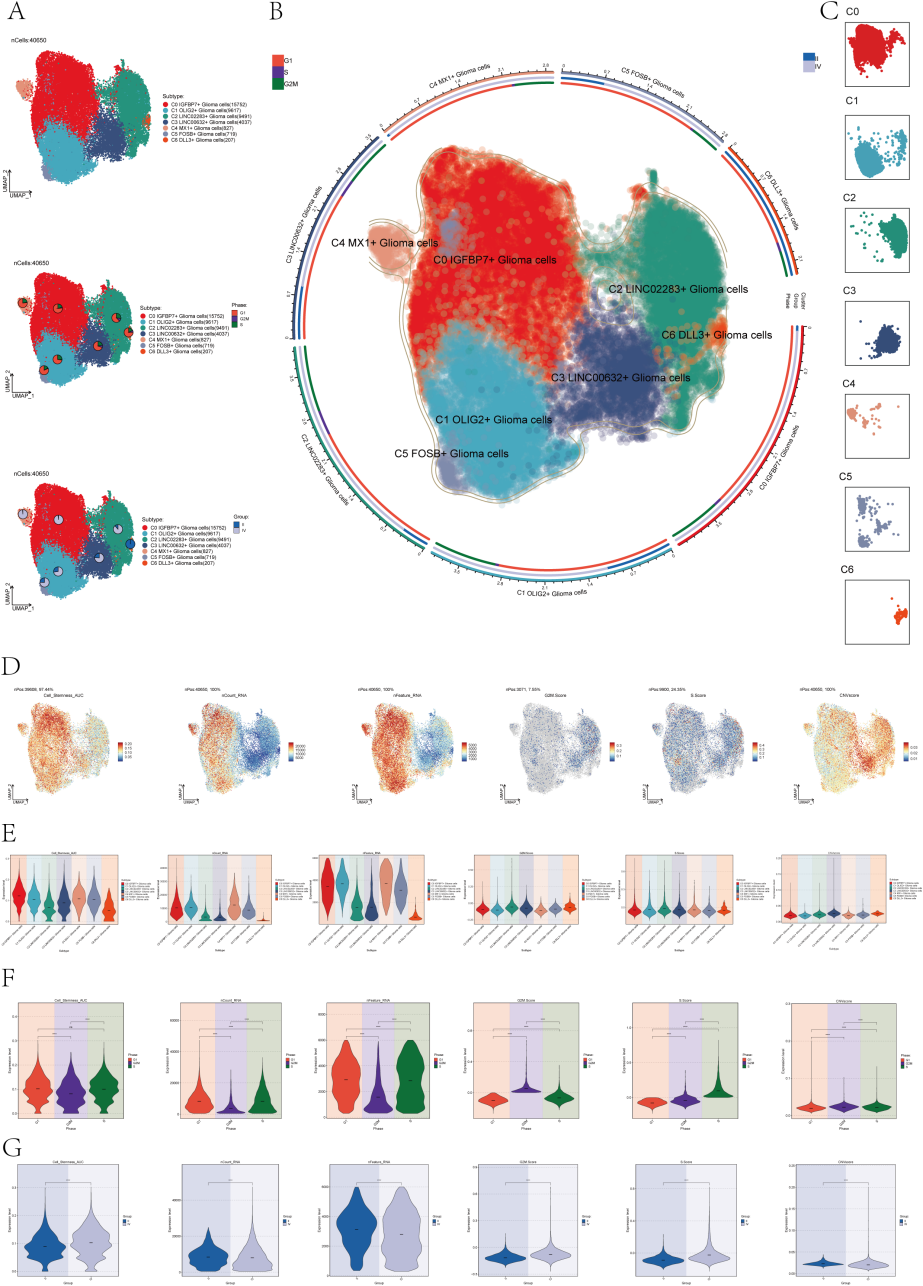
Subcluster identification of astrocytoma. **(A)** The UMAP plot revealed 7 subclusters of 40,650 astrocytoma (top). UMAP visualizations, along with pie graphs, illustrated the breakdown of individual subgroups based on Phases (G1, S, and G2M) (center) and Groups (II and IV) (lower section). **(B)** An integrated visualization demonstrated the distribution of astrocytoma subclusters, phases, and groups. **(C)** UMAP facet map exhibited the distribution of each astrocytoma subcluster. **(D)** UMAP plots individually showcased the Cell Stemness AUC, nCount RNA, nFeature RNA, G2M Score, S Score, and CNV Score of astrocytoma. **(E-G)** Violin plots respectively, displayed the levels of Cell Stemness AUC, nCount RNA, nFeature RNA, G2M Score, S Score, and CNV Score for each astrocytoma subcluster **(E)**, each cell phase **(F)**, and each group **(G)**. Significance levels were denoted as follows: ***P < 0.001, and ****P < 0.0001; NS was used to represent lack of significance.

In order to distinguish malignant cells, we used the InferCNV algorithm to analyze the copy number variation (CNV) level of astrocytes, and the result was shown in **Supplementary Figure 2**. Based on the inferred CNV results, astrocytes with high levels of CNV were defined as tumor cells as astrocytoma. We classified the 40,650 astrocytomas obtained by Seurat and named the seven subclusters according to the marker genes as C0 IGFBP7+ Glioma cells, C1 OLIG2+ Glioma cells, C2 LINC02283+ Glioma cells, C3 LINC00632+ Glioma cells, C4 MX1+ Glioma cells, C5 FOSB+ Glioma cells, and C6 DLL3+ Glioma cells. The 2D map of UMAP dimensionality reduction combined with pie charts showed the distribution of subgroups and their proportion in different cell phases (G1, G2M, and S) and in different groups (II and IV) (**Figure 1A**). The results showed that most of the astrocytoma subclusters had a higher percentage of G1 cell cycle, in addition, C0 IGFBP7+ Glioma cells and C4 MX1+ Glioma cells had a higher percentage of Group IV, suggesting that the malignant degree of cells in these two subclusters might be higher. **Figure 1B** of the of the UMAP diagram showed the distribution of each subgroup and the proportion of cell cycle and group. **Figure 1C** UMAP faceted plots depicting the distribution of each subpopulation in detail.

Next, to dig deeper into the relevant features of astrocytoma, we calculated the Cell Stemness AUC (Area Under the Curve), nCount _RNA, nFeature _RNA, G2M.Calculated the Score, S. Score, and CNV Score for each subgroup and displayed them using UMAP plots (**Figure 1D**). The relevant features of different cellular phases and different groups were demonstrated with violin plots (**Figures 1E–G**). The results showed that C0 IGFBP7+ Glioma cells had the highest cell stemness among the seven subclusters, and C2 LINC02283+ Glioma cells had the highest G2M.Score (**Figure 1E**). In addition, compared with subgroup II, subgroup IV had higher cell stemness and had higher G2M.Score and CNV Score (**Figure 1G**).

### Correlation enrichment analysis

**Figure 2:**
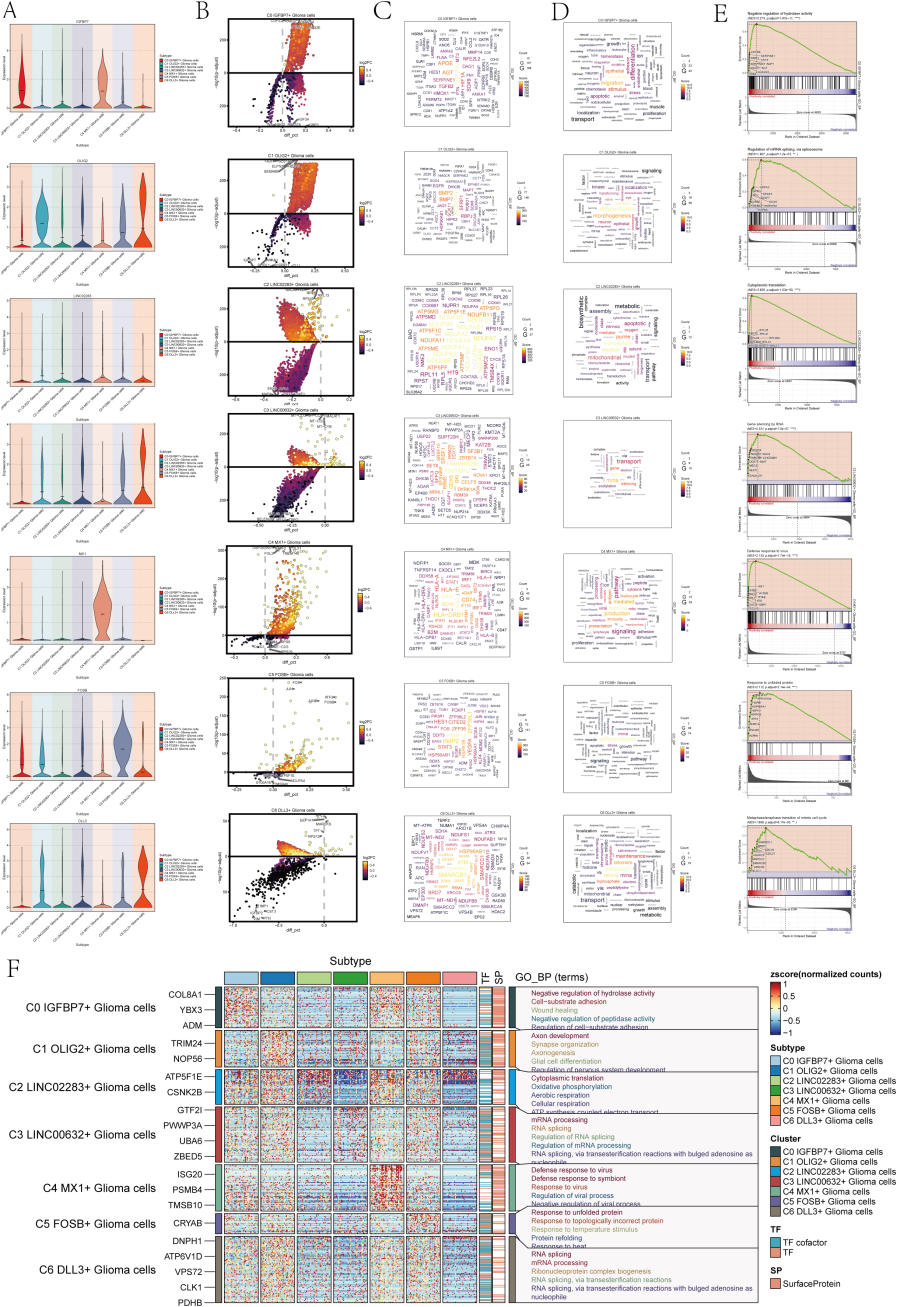
Enrichment analysis of astrocytoma subclusters. **(A)** Violin plots illustrated the distribution of named genes in each subcluster of the 7 astrocytoma subclusters. **(B)** Volcanic plots illustrated the genes with differential expression in the C0-C6 subgroups. **(C)** Cloud diagrams presented the expression patterns of highly-enriched genes in each astrocytoma subcluster. The font size indicated the quantity of genes, while the color indicated the enrichment score for each gene. **(D)** Cloud diagrams displayed the specific enriched pathways of highly-enriched genes in each astrocytoma subcluster. The font size indicated the quantity of genes, while the color indicated the enrichment score of genes within that pathway. **(E)** GSEA enrichment analysis results for each astrocytoma subcluster, showing only the pathway with the highest NES value. **(F)** Heatmap showed the gene expression and top 5 GO-BP enrichment analysis results for each astrocytoma subpopulation.

To comprehend the biological mechanisms linked to each subgroup of astrocytoma, we conducted various enrichment analyses on the distinct genes within the seven subclusters of astrocytoma. **Figure 2A** violin plots demonstrated the expression levels of the named genes of the subclusters in each subcluster, and interestingly, IGFBP7, the named gene of the C0 subcluster, was also expressed in the C4 subcluster. We visualized the DEGs (differential expressed genes) in each subcluster of astrocytoma using volcano plots (**Figure 2B**).

Then, we plotted the gene cloud diagrams for each subgroup of astrocytoma and the cloud diagrams for enrichment analysis according to the number of gene occurrences and the level of enrichment scores of each subgroup, as shown in **Figures 2C, D**.

Furthermore, GSEA was conducted for every subgroup, revealing the pathways with the highest NES values displayed in **Figure 2E**. The top GSEA pathways for these seven subpopulations included negative regulation of hydrolase activity, regulation of mRNA splicing via the spliceosome, cytoplasmic translation, gene silencing by RNA, defense response to viruses, response to unfolded proteins, and metaphase/anaphase transition of the mitotic cell cycle based on the highest NES values.

In addition, in order to visualize the GOBP (Gene Ontology Biological Processes) enrichment analysis of each subpopulation of astrocytoma, we generated a heatmap to show the top 5 enriched terms of each subpopulation (**Figure 2F**).

The findings indicated that the enhanced pathways in C0 IGFBP7+ Glioma cells included inhibiting hydrolase activity, promoting cell-substrate adhesion, aiding in wound healing, inhibiting peptidase activity, and regulating cell-substrate adhesion. This result suggests that the C0 subpopulation may be associated with the adhesion movement of glioma cells. The enrichment pathways of C1 OLIG2+ Glioma cells for axis development, synapse organization, axionogenesis, glial cell differentiation, and regeneration of nervous system development suggest that this subpopulation may be involved in nervous system development and related tissue differentiation. Glioma cells with C2 LINC02283+ Glioma cells were found to have high levels of cytoplasmic translation, oxidative phosphorylation, aerobic respiration, cellular respiration, and ATP synthesis-linked electron transport, indicating a strong connection to cellular respiration and energy metabolism. On the other hand, glioma cells with C3 LINC00632+ Glioma cells showed enrichment in mRNA processing, RNA splicing, regulation of RNA splicing, regulation of mRNA processing, RNA splicing, and via transesterification reactions with bulged adenosine as a nucleophile, suggesting that this subpopulation may play a role in regulating RNA processing.

C4 MX1+ Glioma cells showed enrichment in immune responses to viruses and symbionts, as well as in regulating viral processes and negative regulation. On the other hand, C5 FOSB+ Glioma cells were enriched in responses to protein misfolding, temperature changes, and topologically incorrect proteins. Additionally, these cells also showed enrichment in responses to viruses, symbionts, viral processes, and negative regulation. Response to temperature stimulus, protein refolding, and resistance to heat pathways suggest that the C5 subpopulation may be involved in protein response-related biological processes. C6 DLL3+ Glioma cells are involved in RNA splicing, mRNA processing, and ribonucleoprotein complexes. Ribonucleoprotein complex formation occurs through RNA splicing, involving transesterification reactions and bulged adenosine. The C6 subpopulation may be involved in RNA splicing and other related biological processes through transesterification reactions involving bulged adenosine as a nucleophile.

### Trajectory analysis of the astrocytoma subcluster

**Figure 3:**
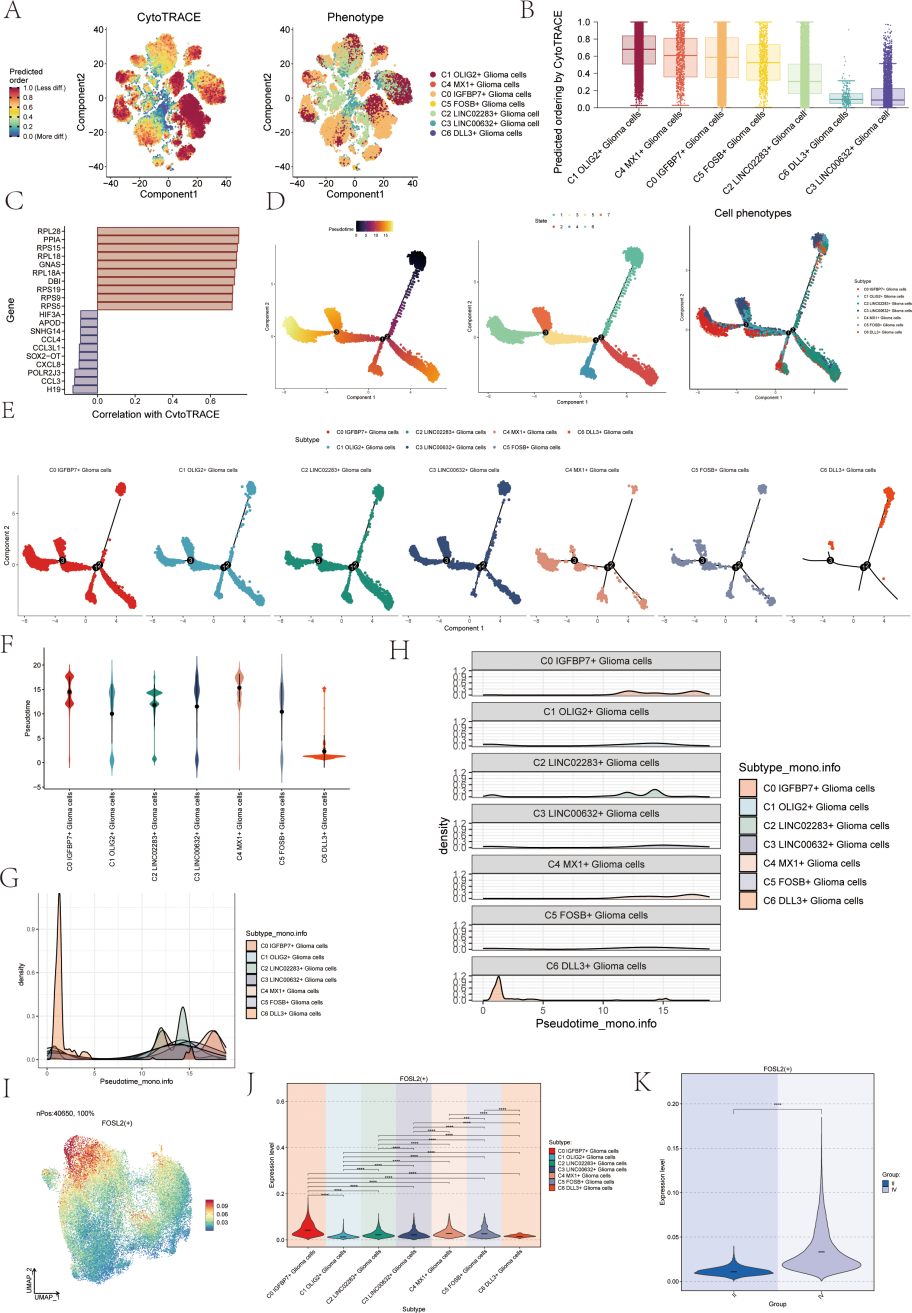
CytoTRACE and Monocle2 pseudotime analysis of astrocytoma subpopulations and related transcription factors. **(A)** CytoTRACE analysis and visualization of the differentiation degree for each astrocytoma subpopulation. In the left figure, dark-green indicated higher differences (low stemness), while dark-red indicated lower differences (high stemness). In the right figure, different colors represent different astrocytoma subpopulations. **(B)** Boxplot displayed the CytoTRACE analysis results, revealing that C1 OLIG2+ Glioma cells, C4 MX1+ Glioma cells, and C0 IGFBP7+ Glioma cells exhibited higher differentiation potential, while C3 LINC00632+ Glioma cells had the lowest differentiation potential. **(C)** Bar graph showed the gene correlations in the CytoTRACE analysis. **(D)** Trajectory analysis using Monocle2, with 3 branch points and 6 states. **(E)** Monocle2 pseudotime analysis facet map depicted the trajectories of the 7 astrocytoma subclusters. **(F)** Violin plots showed the distribution of the 7 astrocytoma subgroups along the pseudotime trajectory. **(G, H)** Ridge plots and their facet maps displayed the density distribution of the 7 astrocytoma subgroups along the pseudotime trajectory. **(I)** UMAP plot visualized the distribution of the top transcription factor (TF) FOSL2 in C0 IGFBP7+ Glioma cells. **(J)** Violin plot presented the distribution of FOSL2 in astrocytoma for each subcluster. **(K)** Violin plot illustrated the distribution of FOSL2 in different groups (II and IV). Significance levels were denoted as follows: **P < 0.01, ***P < 0.001, and ****P < 0.0001; NS was used to represent lack of significance.

To investigate the differentiation status and developmental trajectory of seven subgroups of astrocytoma, we performed CytoTRACE analysis and monocle 2 pseudotime analysis on these subgroups. The related results of the CytoTRACE analysis were shown in **Figures 3A, B**. The CytoTRACE results showed that the CytoTRACE scores of subcluster C1, subcluster C4, and subcluster C0 were higher, indicating that the stemness was higher in these three subclusters. The gene correlations involved in the CytoTRACE analysis can be observed in the bar graph (**Figure 3C**). The findings from the pseudotime analysis of the astrocytoma subgroup were displayed in **Figure 3D**. The findings indicated that the pseudotime path deviated from the top right to the bottom left, encompassing six stages and three points of divergence. The pseudotime facets of the along-trajectory distribution of each subgroup of astrocytoma were shown in **Figure 3E**. In addition, we further demonstrated the pseudotime results of astrocytoma subgroups with Violin plots and ridge plots (**Figures 3F–H**). These results indicated that C0 IGFBP7+ Glioma cells might be at the end of differentiation and have high differentiation ability, and C6 DLL3+ Glioma cells might be at the initial stage of differentiation.

### Transcription factors related to the C0 IGFBP7+ glioma cells subgroup

We analyzed the TOP1 transcription factor FOSL2 of the C0 IGFBP7+ Glioma cells subgroup, which may be at the end of differentiation. Initially, a UMAP visualization was created to display the distribution of the transcription factor FOSL2 (**Figure 3I**), revealing its limited presence in various subgroups. The specific differences in the distribution of transcription factor FOSL2 in each subgroup were shown in **Figure 3J**. The transcription factor FOSL2 was most distributed in the C0 IGFBP7+ Glioma cells subgroup, and the distribution in other subgroups was different, with statistical differences. In addition, the transcription factor FOSL2 was more distributed in highly differentiated tissues (Group IV) than in Group II, and the results were statistically different (**Figure 3K**).

### Slingshot pseudotime analysis of the astrocytoma subcluster

**Figure 4:**
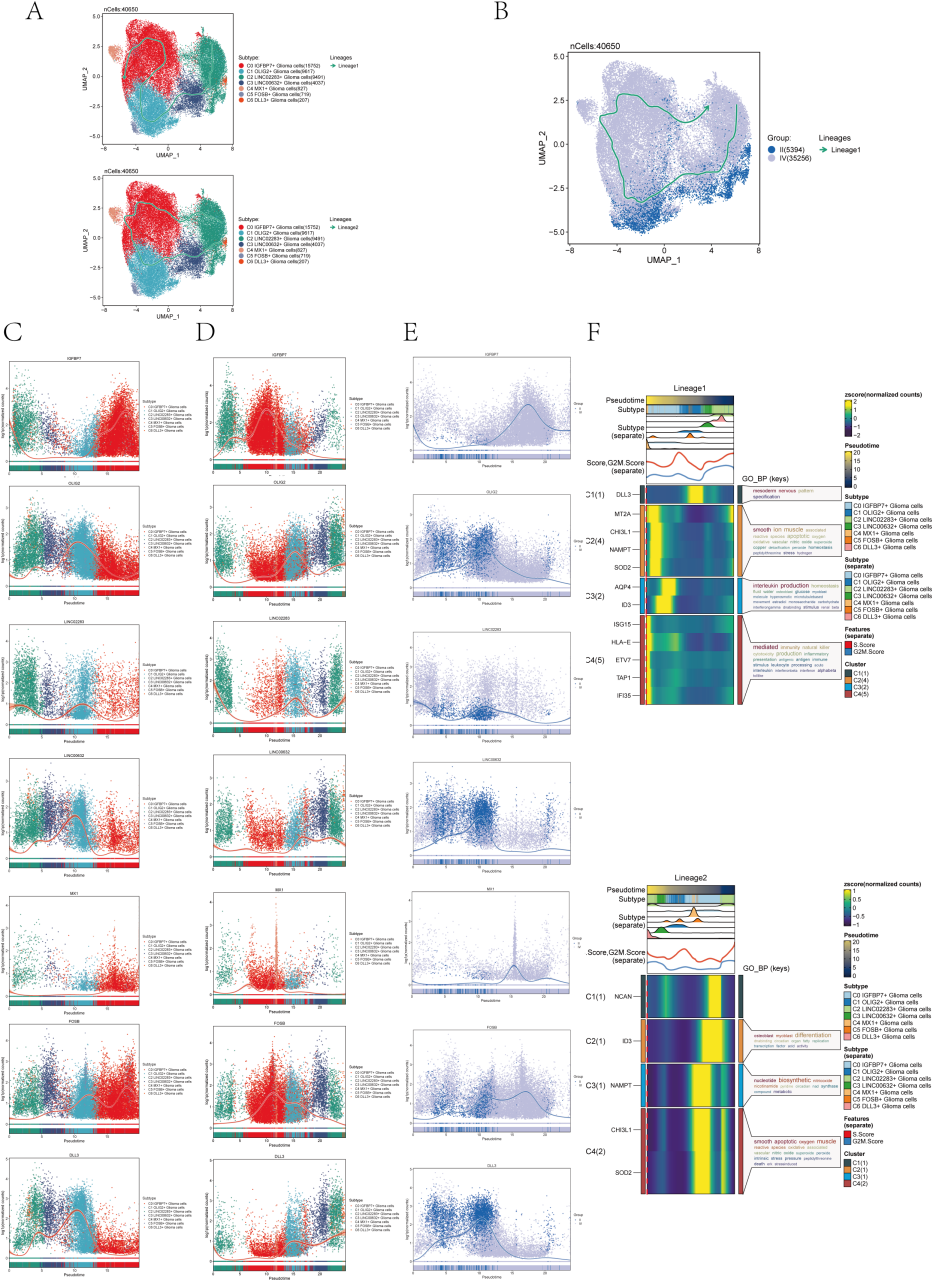
Slingshot pseudotime analysis of astrocytoma. **(A)** Slingshot pseudotime analysis results for the 7 subclusters of astrocytoma reveal 2 lineages. **(B)** Slingshot pseudotime analysis results for different Groups (II and IV) of astrocytoma, showing 1 lineage. **(C)** Scatter plots demonstrated the expression changes of lineage 1-associated genes in the astrocytoma subclusters. **(D)** Scatter plots illustrated the expression changes of lineage 2-associated genes in the astrocytoma subclusters. **(E)** Scatter plots displayed the expression changes of lineage 1-associated genes in the astrocytoma subclusters across the Groups. **(F)** Heatmaps exhibited the expression changes of differentially expressed genes along the trajectories of the 2 lineages of the astrocytoma subclusters, along with their GOBP enrichment analysis results.

In order to further confirm the differentiation relationship between different subgroups of astrocytoma, we conducted a slingshot pseudotime analysis on the astrocytoma subgroup. The findings indicated the presence of two lineages in the slingshot pseudotime assessment of seven subtypes of astrocytoma (**Figure 4A**). Lineage 1 originated from C2 and ends at CO. Lineage 2 originated from C2, passed through CO/C4→C1/C5→C3, and ended with C6. However, there was only one lineage in the slingshot pseudotime analysis of two Groups (II and IV) (**Figure 4B**). The expression of named genes with subpopulation slingshot pseudotime analysis lineage 1 was shown in **Figure 4C** scatter plots, and the expression of named genes with subpopulation slingshot pseudotime analysis lineage 2 was shown in **Figure 4D**. In addition, we also analyzed the trajectories of the slingshot pseudotime analysis of different groups (IV and II), and the slingshot pseudotime analysis of different groups only had lineage 1. The expression of string hot pseudotime analysis lineage 1 with different groups of named genes was shown in **Figure 4E**. The findings indicated that the gene IGFBP7, belonging to the C0 subgroup, exhibited the highest expression levels in Group IV following the slingshot pseudotime analysis. This was consistent with the previous results of CytoTRACE analysis and monocle 2 pseudotime analysis that C0 IGFBP7+ Glioma cells were at the end of differentiation and had high cell stemness, with most of the C0 subclusters distributed in subgroup IV. We also analyzed the expression of two lineages of DEGs with the subgroup’s slingshot pseudotime analysis, and the result was as shown in **Figure 4F**. We also analyzed the enrichment of DEGs by GOBP and found that lineage 1 was mainly enriched with mesodem nervous, smooth ion muscle, DEGs of interleukin production, and mediated, while lineage 2 was enriched with osleoblast, nucleotide biosynthetic, smooth apoptotic, and other pathways.

### Cellular communication network

**Figure 5:**
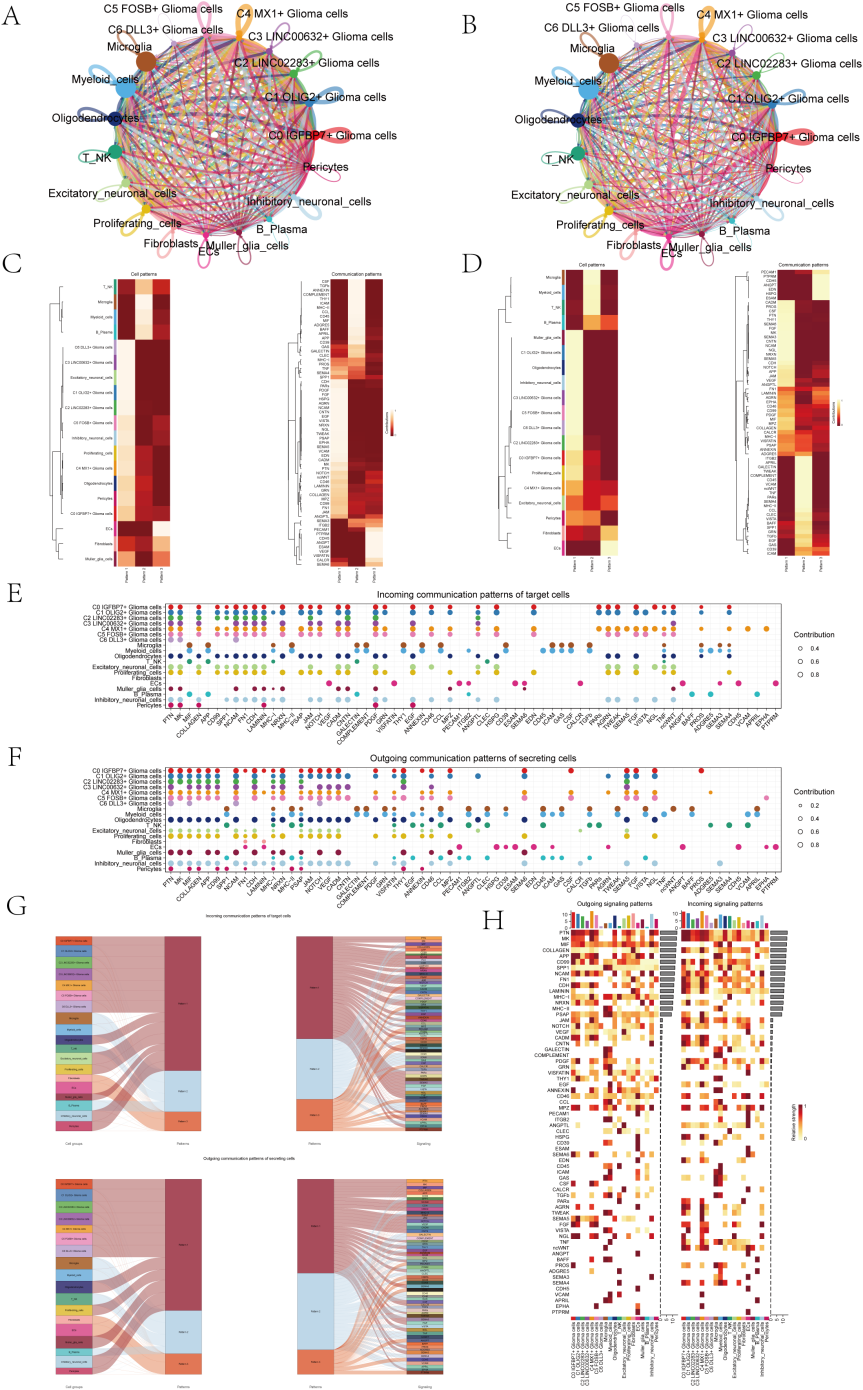
Overview of Cell Communication. **(A)** Weighted interaction network diagram of cellular interactions for all cell types. Thicker lines represented stronger interactions between the cell types. **(B)** Interaction count network diagram of cellular interactions for all cell types. Thicker lines indicated a higher count of interactions between the cell types. **(C, D)** Heatmaps respectively displayed the patterns identified in the incoming communication **(C)** and outgoing communication **(D)**. **(E, F)** Dot plots compared the communication patterns received by target cells **(E)** with the communication patterns sent out by secreting cells **(F)**. **(G)** Sankey charts illustrated the projected communication flow patterns of recipient cells, revealing the coordination between cells receiving signals and their interaction with specific signaling pathways in response (top). In addition, the secretion behaviors of cells were illustrated (bottom), demonstrating how cells interact as message transmitters and how they interact with specific signaling pathways to facilitate communication. **(H)** The heatmap displayed the communication patterns of all cells, showing both outgoing and incoming signals.

In order to systematically explore the interaction of the tumor microenvironment in astrocytoma, we used Cellchat analysis to draw a cell communication network to show the intensity (**Figure 5A**) and quantity (**Figure 5B**) of ligand-receptor interaction between different cell groups. Then, we analyzed the signal patterns between astrocytoma and other cells and the interaction between cells and pathways. Three outgoing signal patterns and three incoming signal patterns were identified, and the results were shown in **Figures 5C, D**. **Figures 5E, F** displayed the communication patterns received by target cells and sent by secreting cells, respectively. The results showed that both C0-C6 subgroups were involved in the PTN signal network pathway.

The results of Sankey diagrams revealed that the cell groups in outgoing patterns were astrocytoma, Oligodendrocytes, Excitatory neuronal cells, Proliferating cells, Muller glia cells, Inhibitory neuronal cells and Pericytes send out signals in coordination with the signal paths PTN, MK and SPP1 belonging to pattern1 (**Figure 5G**).

The heatmap specifically showed (**Figure 5H**) the signal intensity of outgoing signaling patterns and incoming signaling patterns interacting with other cell types.

### PTN signaling network pathway

**Figure 6:**
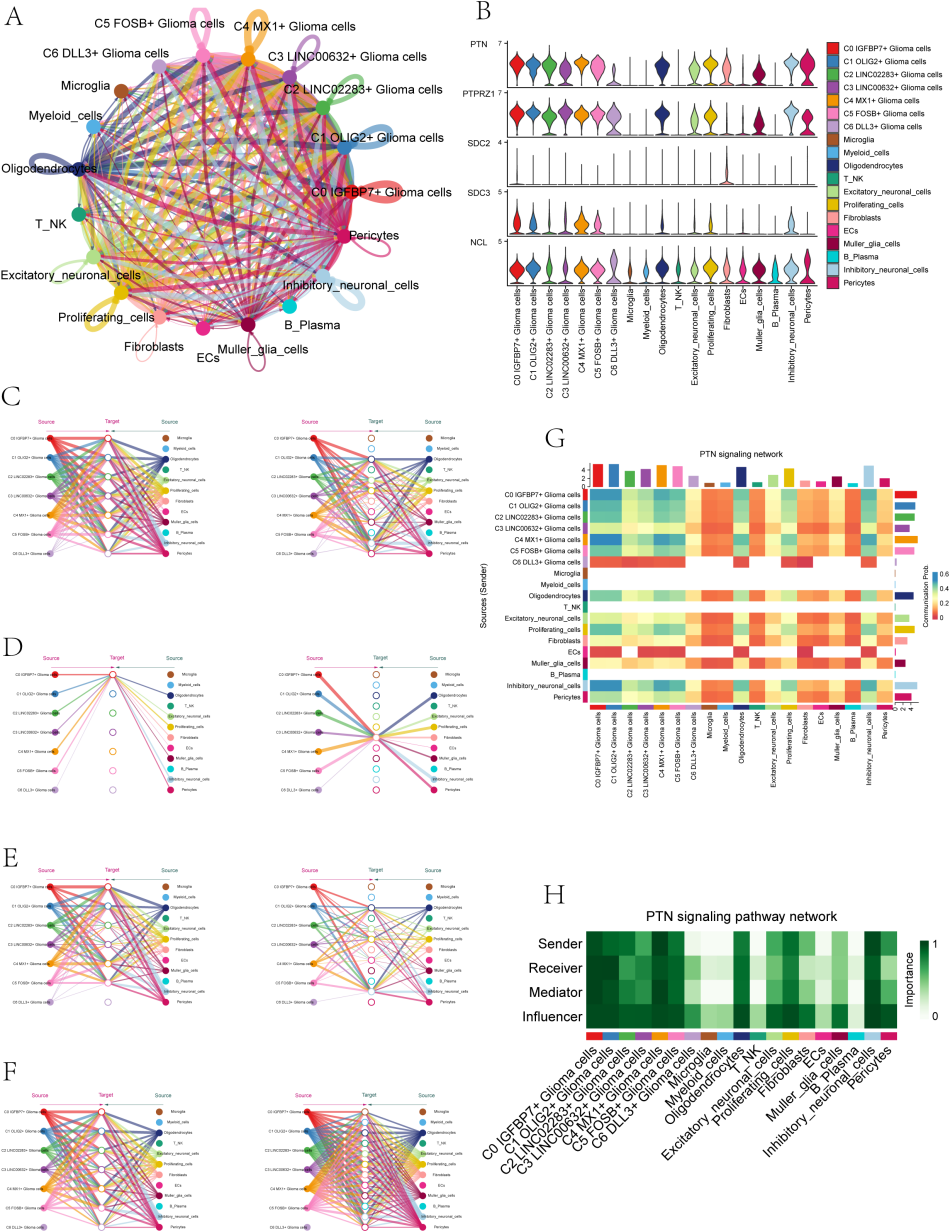
PTN signaling network. **(A)** Circle plot illustrated the interactions of astrocytoma in the PTN signaling network as receiver cells. **(B)** Violin plots displayed the levels of expression of signaling genes related to the PTN signaling network in astrocytoma subgroups and various cell types. **(C-F)** Hierarchical plots depicted the communication networks involving PTPRZ1 **(C)**, SDC2 **(D)**, SDC3 **(E)**, and NCL **(F)** in the inferred PTN signaling network. Source cells were represented by filled circles, while target cell types were represented by open circles. **(G)** The heatmap displayed the calculated four centrality metrics of the PTN signaling network, highlighting the significance of each cell type in terms of sending, receiving, mediating, and influencing.

Because the seven subpopulations of astrocytoma involved the PTN signaling network pathway in both Incoming communication and Outgoing communication, we initiated further studies on the PTN pathway. When glioma cells were used as receiver cells in PTN signaling network pathway, the interaction of various cell-like cells was shown in **Figure 6A**, and the expression levels of signal genes involved in this pathway (PTPRZ1, SDC2, SDC3, NCL) were shown in **Figure 6B**. The results showed that NCL has a certain expression level in various cell-like cells. We further explored the cellular communication networks involved in PTN signaling network. We further explored the PTN signaling network involved in the intercellular communication network, PTPRZ1 Glioma cells, SDC2 Glioma cells, SDC3 Glioma cells, NCL Glioma cells were used as receivers in the respective hierarchical plots shown in **Figure 6C** (PTPRZ1), **Figure 6D** (SDC2), **Figure 6E** (SDC3), and **Figure 6F** (NCL), respectively. The specific interaction between PTN signaling network cells was comprehensively demonstrated by the heatmap (**Figure 6G**). The heatmap (**Figure 6H**) displayed the varying significance of different cell types in the PTN signaling pathway network, acting as senders, receivers, mediators, and influencers. The results showed that in the PTN pathway, six subgroups of malignant asteroids (C0-C5) had high relative importance as sender, receiver, mediator and influencer.

### Establishment and verification of a prognostic model

In order to better serve the clinic, we evaluated the prognostic characteristics of the C0 IGFBP7+ Glioma cell subgroup identified in this study.

**Figure 7:**
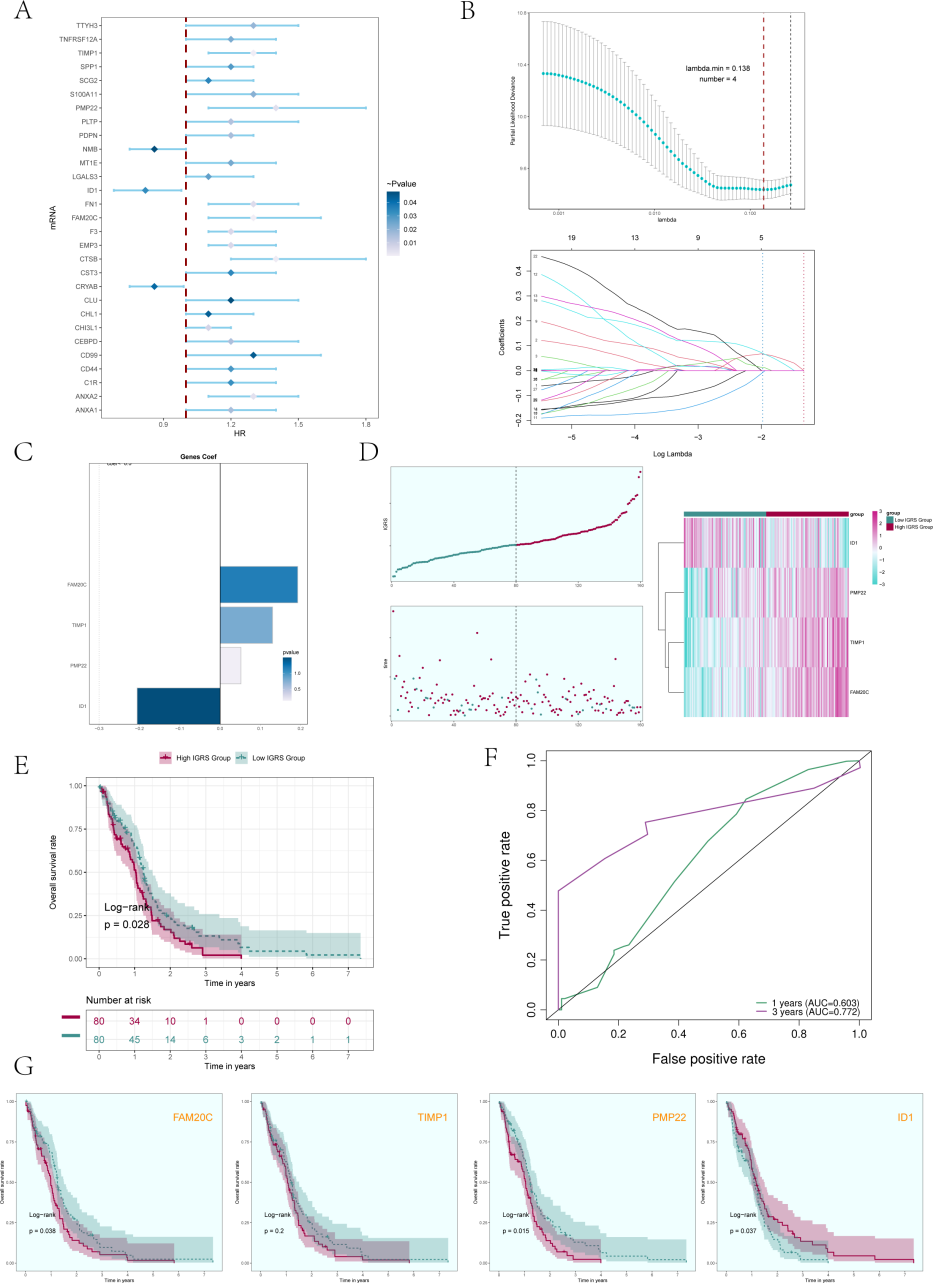
Construction and validation of a prognostic risk model. **(A)** Forest plot presented the results of the univariate Cox analysis (P < 0.05). A HR value less than 1 represented protective genes, whereas a HR value greater than 1 represented risk genes. The color depth represents the magnitude of the p-value. **(B)** The results of the LASSO regression analysis indicated that the optimum lambda value was 0.138, yielding the most favorable outcome. Four genes, namely FAM20C, TIMP1, PMP22, and ID1, had been incorporated into the construction of the risk model. **(C)** Bar graph displaying the Coef values and corresponding p-values for the 4 genes. **(D)** C0 subcluster was divided into High IGRS Group and Low IGRS Group based on the IGFBP7 Risk Score (IGRS). The scoring distribution of the C0 subcluster was displayed in the curve plot (top left), while the survival status of the High IGRS and Low IGRS Groups was shown in the scatter plot (bottom left), and the gene expression patterns contributing to the IGRS were visualized in the heatmap. The color green indicated the Low IGRS Group, while the color red indicated the High IGRS Group. **(E)** Kaplan-Meier analysis findings for the High IGRS Group and Low IGRS Group were presented. **(F)** ROC curves showed the AUC of the risk model for predicting survival at 1 and 3 years. **(G)** Survival plots for the four genes associated with prognosis that make up the IGFBP7 Risk Score.

Initially, we analyzed the leading 100 potential genes in this specific group through univariate Cox regression analysis, revealing that 29 genes were linked to patient prognosis (**Figure 7A**). In order to avoid the multiple contributions of the screened genes, we conducted LASSO regression analysis on these 29 genes (**Figure 7B**), and a total of 4 genes were determined to be significantly related to the prognosis of patients. After screening four genes (FAM20C, TIMP1, PMP22, and ID1), we performed a multivariate Cox regression analysis and identified three genes (FAM20C, TIMP1, and PMP22) as risk factors, with gene ID1 being a protective factor (**Figure 7C**). Using the Cox regression coefficient for each gene, we developed an IGFBP7 Risk Score (IGRS) and determined the IGRS for each sample based on gene expression and the associated coefficient. The specific formula was: IGFBP7 Risk Score (IGRS) = ID1 expression level * (-0.206) + TIMP1 expression level* 0.130 + FAM20C level* 0.192 + PMP22 level* 0.052. According to the score, we divided the C0 IGFBP7+ Glioma cell subgroup into High IGRS Group and Low IGRS Group, and further analyzed the high and low IGRS Groups. The IGFBP7 Risk Score of high and low IGRS Groups and the changes of their living state with time were shown on the left of **Figure 7D**. The expression of four construction model genes in High IGRS Group and Low IGRS Group was shown on the right of **Figure 7D**. The findings indicated that the genes FAM20C, TIMP1, and PMP22 exhibited high expression levels in the High IGRS Group, while the gene ID1 displayed high expression in the Low IGRS Group. Survival analysis comparing high and low IGRS groups indicated that the survival rate was lower in the high IGRS group compared to the low IGRS group (**Figure 7E**). AUC scores for 1 year and 3 years were shown in **Figure 7F**. We analyzed the survival of four modeling genes (FAM20C, TIMP1, PMP22, and ID1) (**Figure 7G**), and the results showed that three genes (FAM20C, PMP22, and ID1) had statistical differences. Among them, the high expression of FAM20C and PMP22 genes has a worse prognosis, while the high expression of gene ID1 has a better survival outcome. Further prove the previous conclusion: genes FAM20C and PMP22 were associated with adverse outcomes.

### Nomogram creation

**Figure 8:**
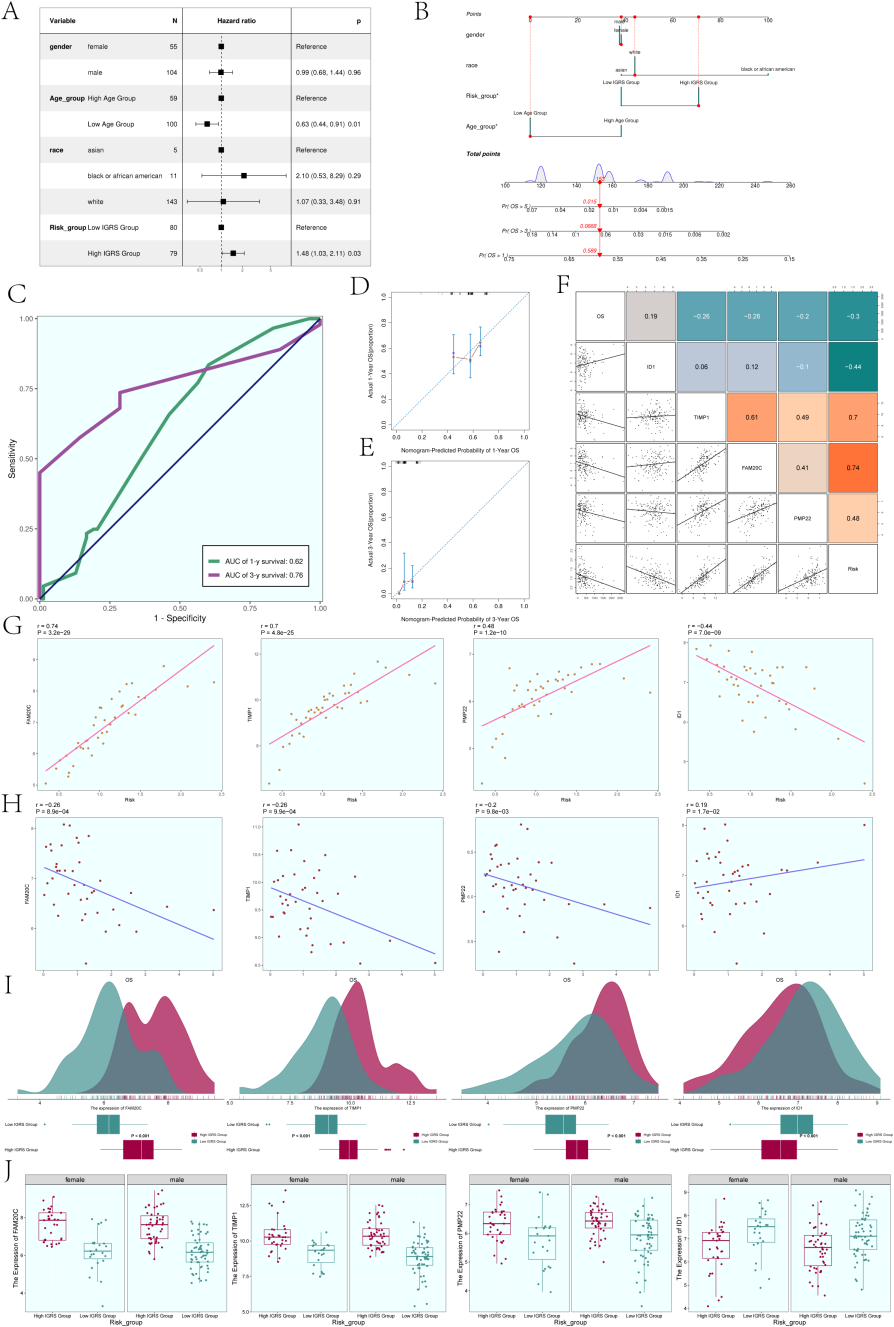
Construction of the Nomogram. **(A)** The forest plot displayed the findings from the multivariate Cox regression analysis, showing that age and IGRS score were identified as separate risk factors. **(B)** Nomogram constructed based on clinical factors (gender, race, age) and the IGFBP7 Risk Score. **(C)** The AUC values for 1-year and 3-year predictions were shown on the ROC curve for the nomogram. **(D, E)** Calibration curves were utilized to evaluate the predictive accuracy of the nomogram for both 1-year and 3-year overall survival (OS). **(F)** Scatter plots combined with a heatmap illustrating the correlations between OS, the four modeling genes, and the IGFBP7 Risk Score. **(G)** Scatter plots demonstrated the correlations between the four modeling genes and the IGFBP7 Risk Score. **(H)** Scatter plots showed the correlations between the four modeling genes and OS. **(I)** Ridge plots combined with box plots displaying the expression levels of the four modeling genes in the High IGRS Group and Low IGRS Group, with both groups sharing the same coordinate system. **(J)** Box plots compared the expression levels of the four modeling genes in the High IGRS Group and Low IGRS Group across different genders. Significance levels were denoted as follows: *P < 0.05; NS was used to represent lack of significance.

In order to further analyze whether IGFBP7 Risk Score can be an independent risk factor, we conducted multivariate Cox regression analysis on clinical factors (gender, age, and race) and IGFBP7 Risk Score (**Figure 8A**). The results of forest plot showed that IGRS Group and IGRS score can be independent prognostic factors.

**Figure 9:**
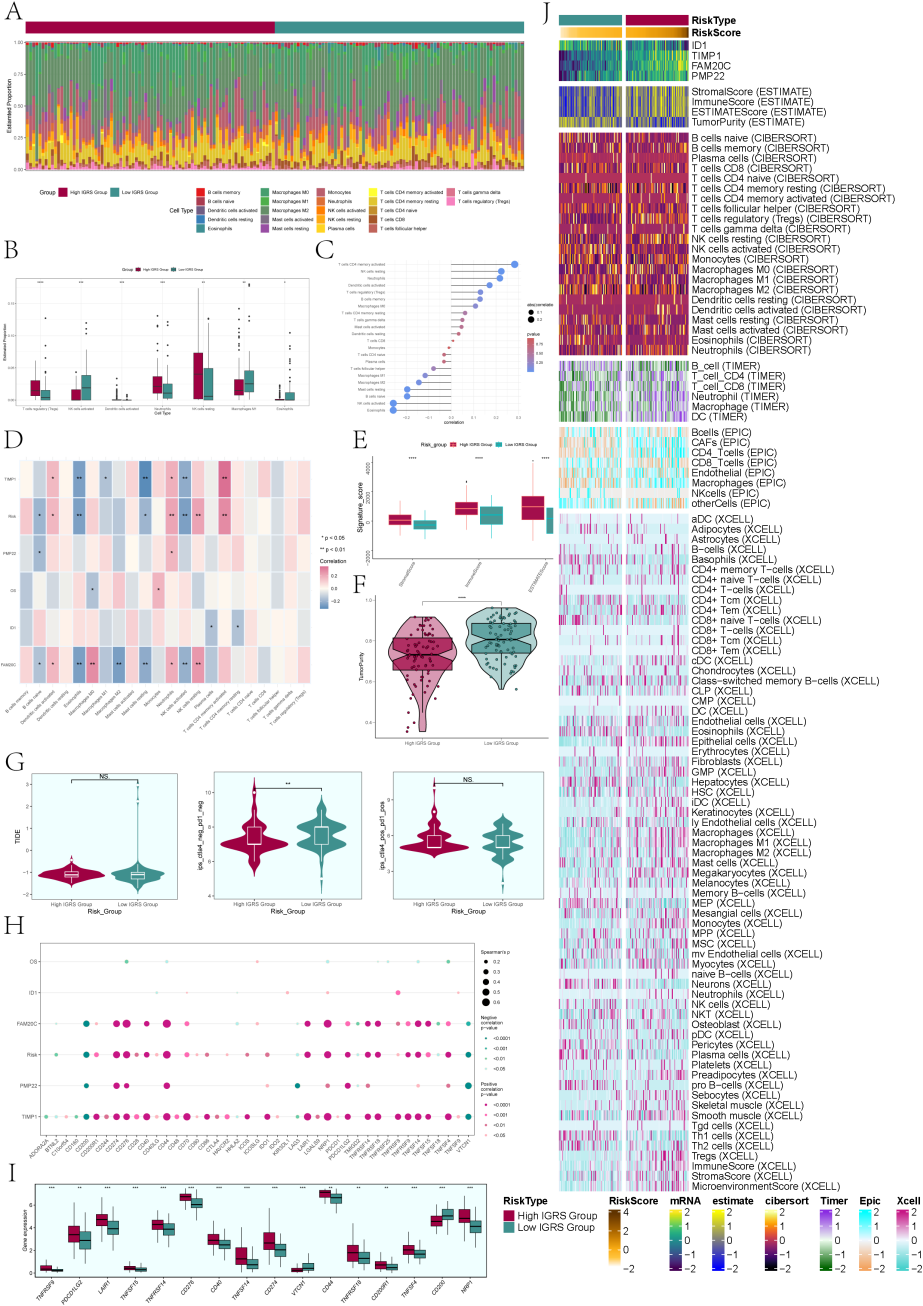
Immune infiltration analysis. **(A)** A heatmap was used to analyze the distribution of 22 immune infiltrating cells between the High IGRS Group and Low IGRS Group. Red represented the High IGRS Group, and green represented the Low IGRS Group. **(B)** Box plot illustrated the distribution of immune infiltrating cells with statistically significant differences between the High IGRS Group and Low IGRS Group. **(C)** Lollipop charts illustrated the relationship between immune infiltrating cells and IGRS. **(D)** The heatmap offered a comprehensive perspective on the relationships among immune infiltrating cells, the four modeling genes, IGRS, and overall survival. **(E)** The box plot illustrated variations in StromalScore, ImmuneScore, and ESTMATEScore between the High IGRS Group and Low IGRS Group. **(F)** Violin plot demonstrated the variations in Tumor Purity levels between the High IGRS Group and Low IGRS Group. **(G)** Violin plots compared the TIDE values and the differences between the ctla4-negative-pd1-negative and ctla4-positive-pd1-positive subgroups in the High IGRS Group and Low IGRS Group. **(H)** The dot plot illustrated the correlations between OS, the four modeling genes, IGFBP7 Risk Score, and immune checkpoint-associated genes. **(I)** Box plot displayed the expression levels of immune checkpoint-associated genes in the High IGRS Group and Low IGRS Group. **(J)** Heatmap provided a comprehensive display of the results from the ESTIMATE, CIBERSORT, EPIC, and Xcell algorithms. Significance levels were denoted as follows: *P < 0.05, **P < 0.01, ***P < 0.001, and ****P < 0.0001; NS was used to represent lack of significance.

In order to determine if the IGFBP7 Risk Score could act as a standalone risk factor, we conducted a multivariate Cox regression analysis that included clinical factors such as gender, age, and ethnicity along with the IGFBP7 Risk Score (**Figure 8B**). The forest plot results suggested that both the IGRS Group and IGRS score may act as separate prognostic factors. **Figure 8C** displayed the AUCs for survival at 1-year and 3-year intervals, while **Figures 8D, E** illustrated the calibration curves for the nomograms at the same intervals, indicating that the nomograms accurately predicted the OS of the training group. **Figure 8F** displayed the pairwise correlation between the four modeling genes, OS, and IGFBP7 Risk Score. The two-by-two correlations between the four modeling genes, OS and IGFBP7 Risk Score were shown in **Figure 9F**. The correlations between the four modeling genes and IGFBP7 Risk Score were visualized with scatter plots (**Figure 8G**), and the results showed that genes FAM20C, TIMP1, and PMP22 were positively correlated with Risk and gene ID1 was negatively correlated with Risk. The correlation analysis of the 4 modeled genes with OS was shown in **Figure 8H**, the results showed that FAM20C, TIMP1, and PMP22 were negatively correlated with OS, while gene ID1 was positively correlated with OS. Then, we further analyzed the specific expression of the four modeled genes in High IGRS Group and Low IGRS Group, and the results were demonstrated by ridge plots combined with box plots (**Figure 8I**). In the High IGRS Group, the genes FAM20C, TIMP1, and PMP22 exhibited increased expression compared to the gene ID1, which was positively correlated with OS. The expression of the four modeled genes was higher in High IGRS Group and Low IGRS Group in different sexes (female and male), as shown in **Figure 8J**.

### Immunoinfiltration analysis of high IGRS group and low IGRS group

To delve deeper into the tumor microenvironment of glioma, we examined the presence of immune cells infiltrating the tumor in both the High IGRS Group and Low IGRS Group of the training cohort, with the findings displayed in a heatmap (**Figure 9A**). The statistically different tumor-immune infiltrating cells were further visualized by box plot (**Figure 9B**), and the evaluation results showed that T cell regulatory (Tregs), Neutrophils, NK cells resting, and Macroghages M1 had higher expression in High IGRS Group, while NK cells activated had higher expression in Low IGRS Group than in High IGRS Group.

To validate the connection between immune cells and IGFBP7 Risk Score in the glioma tumor microenvironment, we assessed the correlation between immune cells and IGRS, presenting the findings through Lollipop plots depicted in **Figure 9C**. We thoroughly analyzed the relationship between immune cells and the four genes that make up IGRS, IGFBP7 Risk Score, and OS and displayed the findings using a heatmap (**Figure 9D**). The findings indicated an inverse relationship between IGRS Score and B cells naive, Eosinophils, Master cells Resting, and NK cells activated, while showing a positive correlation with Dendritic cells activated, Monocytes, NK cells Resting, and T cells CD4 memory Resting. It was worth noting that gene TIMP1 and gene FAM20C were negatively correlated with Eosinophils, Master Cells Resting and NK Cells Activated.

Next, we delved deeper into the variations in Stromal Score, Immune Score, Estmate Score, and Tumour Purity between the High IGRS Group and Low IGRS Group, finding statistically significant differences (**Figures 9E, F**). The Stromal Score, Immune Score, and Estmate Score were elevated in the High IGRS Group, whereas the Tumor Purity was increased in the Low IGRS Group. Nonetheless, there was no statistically significant difference in Tumor Immune Dysfunction and Exclusion (TIDE) between the two groups, suggesting that tumor immune dysfunction and exclusion were similar in both groups (**Figure 9G**). In the study, it was found that the gene TIMP1 exhibited a strong positive correlation with the majority of immune checkpoint-related genes, while the gene ID1 did not show any significant correlation with most immune checkpoint-related genes (**Figure 9H**).

Furthermore, we analyzed the variations in expression of immune checkpoint-associated genes between the High IGRS Group and Low IGRS Group, creating box plots to illustrate the genes exhibiting significant differences (**Figure 9I**). The results of the analysis indicated that the majority of genes associated with immune checkpoints exhibited increased levels of expression in the High IGRS Group, whereas VTCN1 and CD200 displayed higher expression levels in the Low IGRS Group. We used ESTIMATE, CIBERSORT, EPIC, and Xcell algorithms to analyze and display the variations in immune infiltrating cells, Stromal Score, Immune Score, and Tumor Purity between the High IGRS Group and Low IGRS Group in a heatmap. (**Figure 9J**)

### Differentially expressed genes and their enrichment analysis in high and low IGRS groups

**Figure 10:**
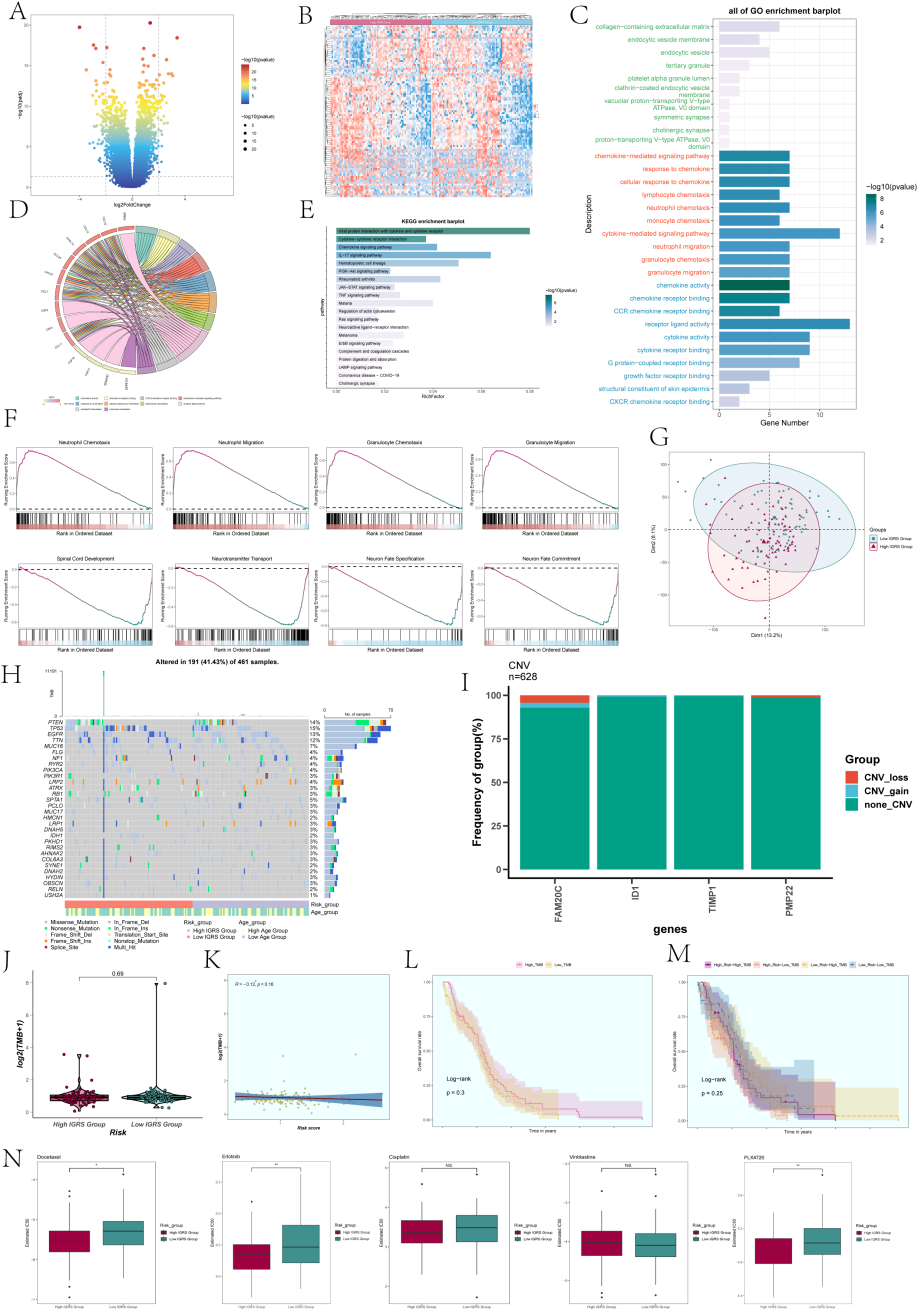
Differentially expressed genes and enrichment analysis in High and Low IGRS Groups. **(A)** The volcano plot displayed the genes that were expressed differently between the High IGRS Group and Low IGRS Group. **(B)** Heatmap depicted the distribution of DEGs in the High IGRS Group and Low IGRS Group. **(C)** Bar graph presented the results of the GOBP, GOCC, and GOMF enrichment analyses for the DEGs. **(D)** Chord plot displayed the relevant genes involved in the GO enrichment analysis items. **(E)** The bar graph displayed the findings of the KEGG examination for the differentially expressed genes. **(F)** GSEA enrichment analysis results for the DEGs, displaying the enrichment scores of genes on different pathways. **(G)** Principal Component Analysis (PCA) plot showing the gene expression clustering distribution differences between the High IGRS Group and Low IGRS Group. **(H)** A waterfall chart displayed the 30 most mutated genes in the High IGRS Group and Low IGRS Group. **(I)** Bar graph displayed the copy number variation status of the four modeling genes, with blue indicating chromosomal copy number increase, red indicating chromosomal copy number decrease, and green indicating no change in chromosomal copy number. **(J)** A box plot displayed the Tumor Mutation Burden (TMB) for both the High IGRS Group and Low IGRS Group. **(K)** The scatter plot displayed the relationship between Tumor Mutation Burden and IGFBP7 Risk Score. **(L)** Kaplan-Meier analysis demonstrated variations in prognosis between High TMB and Low TMB groups. **(M)** Kaplan-Meier survival analysis findings for the High Risk-High TMB, High Risk-Low TMB, Low Risk-High TMB, and Low Risk-Low TMB groups. **(N)** Box plots showed the findings of drug response analysis for the High IGRS Group and Low IGRS Group. Significance levels were denoted as follows: *P < 0.05, **P < 0.01, and NS was used to represent lack of significance.

To compare the High IGRS Group and Low IGRS Group, we computed and studied the DEGs in both groups, presenting them using a volcano plot (**Figure 10A**) and showcasing the specific expression of these DEGs in the groups through a heatmap (**Figure 10B**). Immediately after that, we performed multiple enrichment analyses on these differentially expressed genes. Enrichment analyses were conducted on them, which included examining GOBP (Gene Ontology Biological Processes), GOCC (Gene Ontology Cellular Components), and GOMF (Gene Ontology Molecular Functions). The findings indicated that differentially expressed genes (DEGs) were highly concentrated in functions related to binding between receptors and ligands, signaling pathways mediated by cytokines, and activities involving chemokines (**Figure 10C**). The related genes of the enriched entries are shown in the chord plot (**Figure 10D**). The analysis of enriched pathways using KEGG for the identified DEGs (**Figure 10E**) indicated a significant enrichment in pathways related to viral protein interaction with cytokines and cytokine receptors, interactions between cytokines and cytokine receptors, signaling pathways for chemokines, the IL-17 signaling pathway, and more. According to the findings of GSEA (Gene Set Enrichment Analysis) (**Figure 10F**), the High IGRS Group exhibited increased activity in pathways related to Neutrophil Chemotaxis, Neutrophil Migration, Granulocyte Chemotaxis, and Granulocyte Migration, while showing decreased activity in pathways associated with Spinal Cord Development, Neurotransmitter Transport, Neuron Fate Specification, Neuron Migration, and Neuron Fate Commitment.

PCA was utilized to examine the diversity of gene expression patterns in the High IGRS Group and the Low IGRS Group, with PCA 1 and PCA 2 visualized through scatter plots. PCA 1 and PCA 2 exhibited variances of 13.2% and 8.1%, respectively, as shown in **Figure 10G**. Furthermore, we investigated the somatic gene mutations in both cohorts and highlighted the distinctions among the top 30 genes exhibiting the greatest mutation rates in each group. Variations among 12 genes across various groups indicated that the PTEN gene had the highest mutation frequency, as depicted in **Figure 10H**. Next, we assessed the gene model’s chromosome copy number variation (CNV) and presented the findings using a bar graph (**Figure 10I**). The findings indicated that genes ID1 and TIMP1 did not exhibit any CNV loss or CNV gain, while gene FAM20C experienced both CNV loss and CNV gain events.

A comparison analysis was performed on the two groups’ tumor mutation burden (TMB). The results revealed no statistically significant difference in TMB between the two groups (**Figure 10J**). The correlation analysis between TMB and Risk Score was shown in **Figure 10K**, with an R value of -0.12 and a corresponding p-value of 0.16. Using the TMB as a basis, the participants were separated into two groups, High TMB and Low TMB, for examination of survival rates (**Figure 10L**). Furthermore, the participants were divided into four groups based on their risk level and tumor mutational burden (TMB): High Risk-High TMB, High Risk-Low TMB, Low Risk-High TMB, and Low Risk-Low TMB, which was then followed by an analysis of survival rates. Nevertheless, the findings indicated that there was no notable variation between the groups in terms of statistical significance (**Figure 10M**).

### Drug sensitivity analysis

Analysis of drug sensitivity was performed on the High IGRS Group and Low IGRS Group, showing that Docetaxel had a lower IC50(semi-inhibitory concentration) in the High IGRS Group, as illustrated in **Figure 10N**.Conversely, PLX4720 demonstrated a lower IC50 value in the Low IGRS Group.

### *In vitro* experimental validation

**Figure 11:**
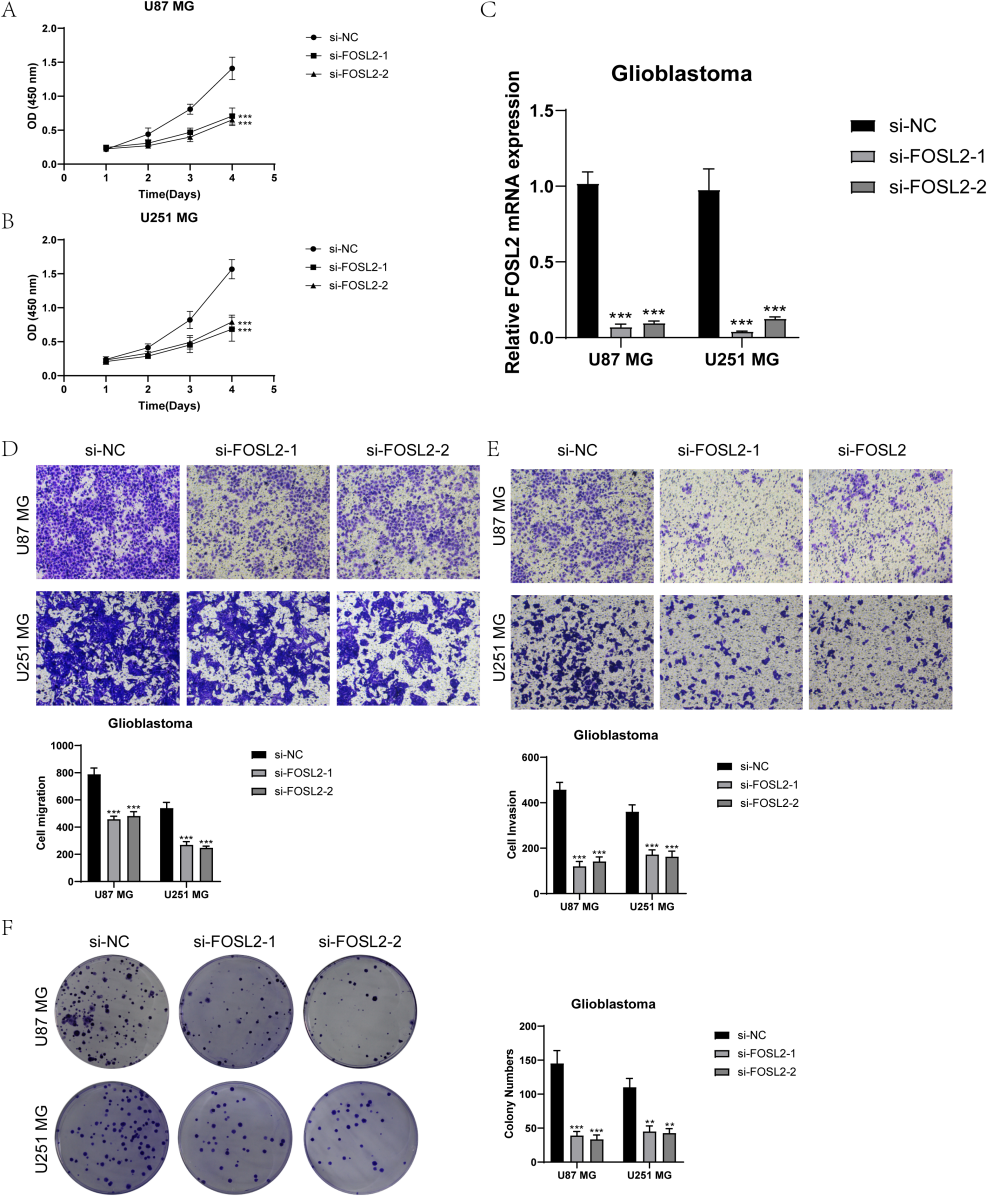
*In vitro* experimental validation. **(A, B)** The CCK-8 assay results showed a notable reduction in cell viability in the U87 MG and U251 MG cell lines following the knockdown of FOSL2. **(C)** The qPCR findings showed the initial levels of FOSL2 mRNA expression in the U87 MG and U251 MG cell lines, as well as the changes in FOSL2 mRNA expression following FOSL2 knockdown. **(D, E)** The transwell test showed that reducing FOSL2 expression greatly hinders the movement and infiltration capabilities of the U87 MG and U251 MG cell lines. **(F)** The plate cloning experiment showed a notable reduction in colony formation capacity in the U87 MG and U251 MG cell lines following the suppression of FOSL2. Significance levels were set at **P < 0.01, and ***P < 0.001.

For further elucidation of the functionality of FOSL2, we conducted *in vitro* functional assessments. Two cell lines, U87 MG and U251 MG, were chosen for comparison with FOSL2 knockdown by establishing a negative control group. The cell activity test (**Figures 11A, B**) showed a notable reduction in cell viability after FOSL2 knockdown, as revealed by the results of the CCK-8 assay. For accuracy, we quantified the levels of FOSL2 mRNA expression in the U87 MG and U251 MG cell lines in both the control and FOSL2 knockdown groups (**Figure 11C**). The transwell test findings showed a significant decrease in the movement and infiltration of U87 MG and U251 MG cells following the suppression of FOSL2 in comparison to the control group (**Figures 11D, E**). Furthermore, the plate cloning results revealed a significant suppression in colony formation quantity after FOSL2 knockdown in both cell line models (**Figure 11F**).

**Figure 12:**
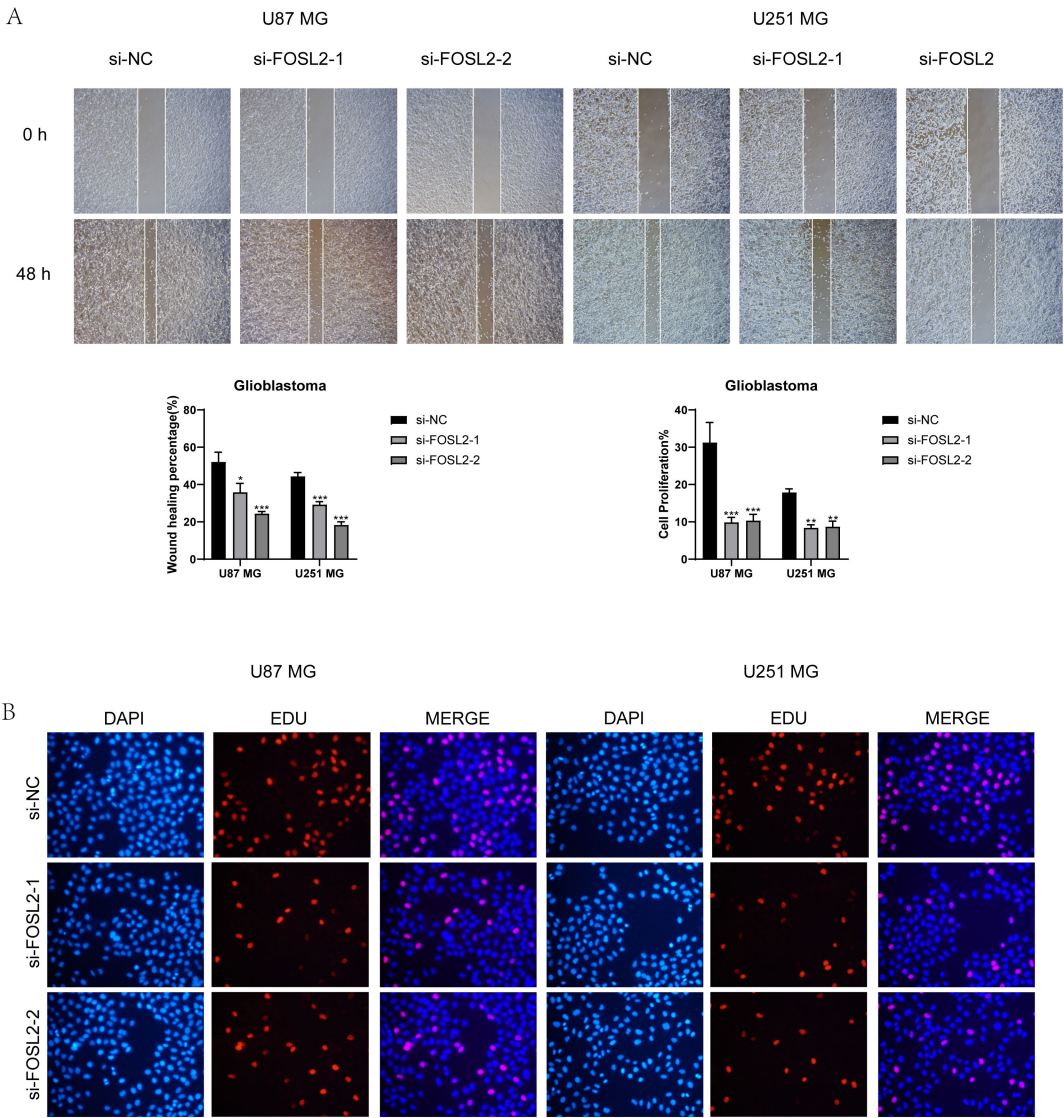
Scratch assay and EdU staining results. **(A, B)** The scratch assay showed that FOSL2 knockdown significantly decreased the movement and infiltration of the U87 MG and U251MG cells. **(C, D)** EdU staining demonstrated that FOSL2 knockdown was shown to inhibit the growth of U87 MG and U251MG cells. Significance levels were set at *P < 0.05, **P < 0.01, and ***P < 0.001.

A healing experiment was performed, revealing a notable increase in the width of the 48-hour scratch in both cell lines after FOSL2 knockdown compared to the negative control group. This suggests a reduction in cell migration rate, supported by statistically significant findings (**Figures 12A, B**). Additionally, EdU staining once again confirmed the decreased proliferative capacity of tumor cells after FOSL2 knockdown (**Figures 12C, D**). Thus, from the above tests, it was noted that reducing FOSL2 results in lower cell proliferation, migration, and invasion in U87 MG and U251 MG cell lines, indicating that FOSL2 could enhance glioma advancement.

## Discussion

Astrocytoma tumors start in the glial cells called astrocytes. The most aggressive astrocytoma is a glioblastoma. Glioblastomas are the most aggressive and lethal brain tumors (53), being the most aggressive and deadly brain tumor with a high likelihood of recurrence and spreading to other areas of the brain (54). To further investigate the internal heterogeneity of glioma, we analyzed glioma single-cell RNA sequencing (scRNA-seq) data to identify the various cell types present, including microglia, oligodendrocytes, astrocytes, inhibitory neuronal cells, and pericytes, among others. Astrocytoma tumors originate in astrocytes. The most aggressive form of astrocytoma is glioblastoma. Additionally, astrocytes encompass the most abundant cellular entities within the central nervous system (55),so we used astrocytes as the main subpopulation of the study. By inferCNV analysis, we defined high levels of astrocytes as astrocytoma, analyzed them by dimensionality reduction clustering, and finally divided them into seven different cell subpopulations: C0 IGFBP7+ Glioma cells, C1 OLIG2+ Glioma cells, C2 LINC02283+ Glioma cells, C3 LINC00632+ Glioma cells, C4 MX1+ Glioma cells, C5 FOSB+ Glioma cells, and C6 DLL3+ Glioma cells. CytoTRACE and Monocle 2 analyses suggested that C0 IGFBP7+ glioma cells were likely at advanced stages of differentiation with high differentiation potential. Since astrocytomas often showed that higher malignancy could correlate with greater differentiation, identifying these cells might have been crucial. They could provide important insights into tumor progression and resistance, potentially guiding more effective treatments.

In order to delve deeper into the connections between the astrocytoma subcluster and various cell types, we employed CellChat analysis. This tool can deduce and examine intercellular communication networks based on single-cell sequencing data, forecasting the primary signals exchanged between cells and how they work together to carry out their functions (56). By analyzing afferent and efferent signals between subclusters of astrocytoma and other cells, it was found that all 7 subclusters of astrocytoma were involved in the PTN signaling network pathway in both Incoming communication and Outgoing communication. Previous research data has indicated that blocking the PTN pathway may serve as a means to combat glioblastoma (57). Disrupting the PTN receptor PTPRZ1 has been shown to inhibit the growth of glioblastoma stem cells (GSCs) (58). Therefore, we conducted further analysis of the PTN pathway and discovered that PTPRZ1 exhibits high expression in various subclusters of astrocytoma. When PTPRZ1 Glioma cells acted as receivers, the subclusters of astrocytoma showed a strong association with other cell types. Furthermore, in the PTN signaling pathway network, the C0 IGFBP7+ Glioma cells subcluster showed greater importance as a sender, receiver, mediator, and influencer when compared to other types of cells. Therefore, we hypothesized that the C0 subgroup was essential in the PTN pathway and impacted the advancement of glioblastoma via this pathway.

To assess the role of the C0 IGFBP7+ glioma cell subgroup in neuroglioma progression, we performed univariate Cox and LASSO regression analyses on candidate genes, identifying four genes strongly linked to prognosis. We developed a prognosis model based on these genes and established the IGFBP7 Risk Score (IGRS). This score classified the training cohort into High IGRS and Low IGRS groups, with survival analysis showing poorer outcomes for the High IGRS group. A nomogram incorporating clinical data and multivariate Cox regression confirmed the IGRS as a standalone predictor of patient outcomes. Analysis of the four genes revealed their distribution and correlation with Risk Score and overall survival. In summary, the IGFBP7 Risk Score (IGRS) provided a robust prognostic tool for astrocytomas by categorizing patients into High and Low IGRS groups, with High IGRS correlating with worse outcomes. It integrated gene expression data to offer improved predictions of patient survival and highlighted key genes like FAM20C and PMP22 associated with poor prognosis. FAM20C has been proven to be a marker of glioma invasion and can be used as a new therapeutic target for GBM (59). However, there are few studies on the relationship between PMP22 and glioma, which need to be further explored.

GBM is a highly immunosuppressive tumor. At present, there is no FDA-approved immunotherapy for glioblastoma (60). We further discussed the relationship between IGFBP7 Risk Score (IGRS) and the immune microenvironment of glioma and analyzed the tumor immune infiltration of the two groups based on High IGRS Group and Low IGRS Group. Compared with Low IGRS Group, T cell regulation (tregs), neutrophils, NK cells resting, and macroghages M1 in High IGRS Group have higher expression, while NK cells activated have higher expression in Low IGRS Group, which was related to the relationship between TME and immune cells in tumors (61).

ESTIMATEScore is calculated by adding ImmuneScore and StromalScore, which indicate the presence of immune or matrix components in the TME (62). The Stromal Score, Immune Score, and EstmateScore were higher in the High IGRS Group, which is intriguing. Furthermore, an examination of somatic cell mutation frequency revealed that the PTEN gene exhibited the highest mutation rate, and PTEN could suppress the activation of the PI3K/AKT/mTOR signaling pathway (63). When the functionality of PTEN is disrupted, such as through mutations in the PTEN gene, it leads to the loss of PTEN’s tumor suppressor capabilities.

Finally, the IC50 (semi-inhibitory concentration) of PLX4720, Docetaxel, and Erlotinib in different groups was analyzed. Docetaxel, an FDA-approved medication, is now the primary therapy for various cancer forms, such as prostate cancer (64) and non-small cell lung cancer (NSCLC) (65). while Erlotinib, a tyrosine kinase inhibitor, is effective against lung cancer, head and neck squamous cell carcinoma (66, 67), and various other types of cancer. The analysis revealed that Docetaxel and Erlotinib had reduced IC50 values in the High IGRS Group, indicating improved efficacy of these drugs for this patient cohort. Consequently, Docetaxel and Erlotinib demonstrate greater therapeutic potential for patients in the High IGRS Group.

Analysis of the transcription factors in the C0 subgroup revealed that the distribution of the transcription factor FOSL2 of TOP1 in Group IV was greater than in Group II. Hence, we performed *in vitro* tests to support the role of crucial transcription regulators. The findings indicated that suppressing FOSL2 can decrease the growth, movement, and infiltration of U87 MG and U251 MG cells, aligning with the findings of Yiyun Chen and Ranhuo et al. (68). Thus, FOSL2 has the ability to enhance the invasion and advancement of gliomas.

However, there are some limitations to this study. First of all, the sample size is small, and the number of patients with glioma obtained in this study is limited. Secondly, we have only done scrna-seq and bulk RNA-seq analyses and *in vitro* experiments, and we need large sample and multi-center research to further explore the relationship between IGFBP7, FOSL2, the IGFBP7 Risk Score (IGRS), and glioma. Therefore, we plan to carry out various analytical methods, such as metabonomics and ATAC-seq, to demonstrate in many aspects.

Nevertheless, there are certain constraints to this research. First of all, the sample size is small, and the number of patients with glioma obtained in this study is limited. Additionally, our research has been limited to scRNA-Seq and bulk RNA-seq analyses along with *in vitro* experiments. To delve deeper into the connection between IGFBP7, FOSL2, the IGFBP7 Risk Score (IGRS), and glioma, we require extensive sample sizes and collaboration with multiple research centers. Therefore, we plan to carry out various analytical methods, such as metabonomics and ATAC-seq, to demonstrate this in many aspects.

## Conclusion

Our exploration of the astrocyte tumor microenvironment highlighted the critical role of the C0 IGFBP7+ glioma subpopulation in astrocytoma progression. We developed the IGFBP7 Risk Score (IGRS) as an independent prognostic tool that effectively separates High and Low IGRS groups, with High IGRS indicating worse outcomes. The IGRS not only predicts patient survival but also identifies key genes like FAM20C and PMP22 linked to poor prognosis. Our study also pinpointed new therapeutic targets, showing that Docetaxel and Erlotinib are more effective in the High IGRS group. Additionally, *in vitro* tests confirmed that transcription regulators like FOSL2 enhance glioma invasion and progression. These insights improve our understanding of astrocytoma and offer promising avenues for future treatments.

## Data Availability

The original contributions presented in the study are included in the article/**Supplementary Material**. Further inquiries can be directed to the corresponding author/s.

## Supplementary material

Supplementary Figure 1 Visualization of large groups of Glioma. **(A)** The UMAP visualization displayed how the different cell types were distributed among 234,148 high-quality glioma cells. **(B-D)** UMAP visualizations, along with pie graphs, illustrated how 234,148 high-grade glioma cells were spread out among Categories (II, III, and IV) **(B)**, Phases (G1, S, and G2M) **(C)**, and 45 seurat clusters **(D)**. **(E)** The bubble chart displayed the top five genes for the 13 types of cells and illustrated how these genes were distributed among the various groups.

Supplementary Figure 2 Analysis of astrocytes through inferCNV. **(A)** The inferCNV analysis of astrocytes was visualized in a heatmap. Astrocytes with high levels of inferCNV were defined as astrocytoma. The red color represented high copy number variation (astrocytoma), while the blue color represented low copy number variation.
