# Supplementary figures and images for: IGFBP7+ subpopulation and IGFBP7 risk score in astrocytoma: insights from scRNA-Seq and bulk RNA-Seq
Source: Front Immunol. 2024 Sep 30;15:1434300. doi: 10.3389/fimmu.2024.1434300 (PMC11471593; doi:10.3389/fimmu.2024.1434300)

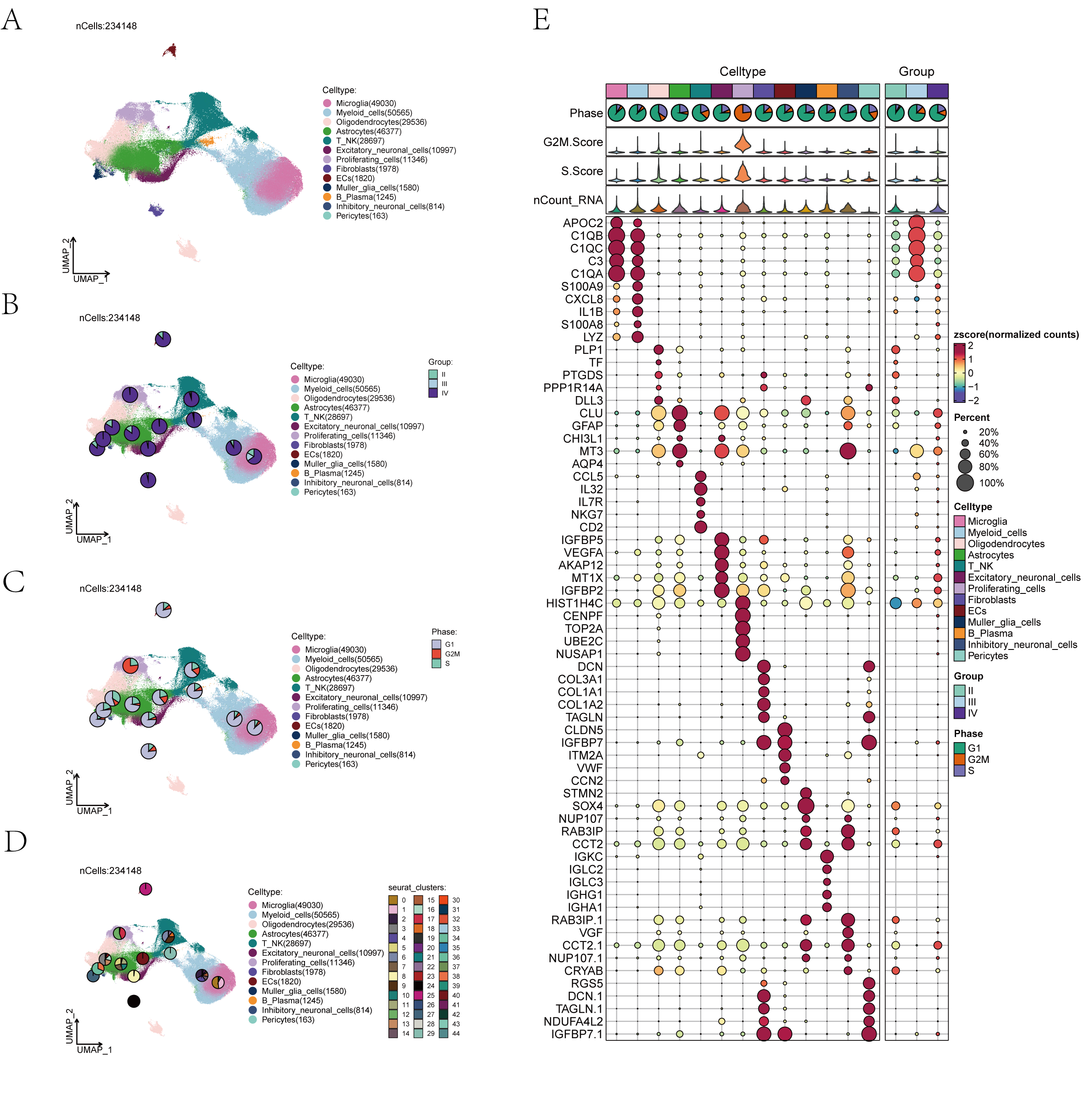

Supplement: Supplementary Figure 1 — Visualization of large groups of Glioma. (A) The UMAP visualization displayed how the different cell types were distributed among 234,148 high-quality glioma cells. (B-D) UMAP visualizations, along with pie graphs, illustrated how 234,148 high-grade glioma cells were spread out among Categories (II, III, and IV) (B), Phases (G1, S, and G2M) (C), and 45 seurat clusters (D). (E) The bubble chart displayed the top five genes for the 13 types of cells and illustrated how these genes were distributed among the various groups. [file Image1.tif]

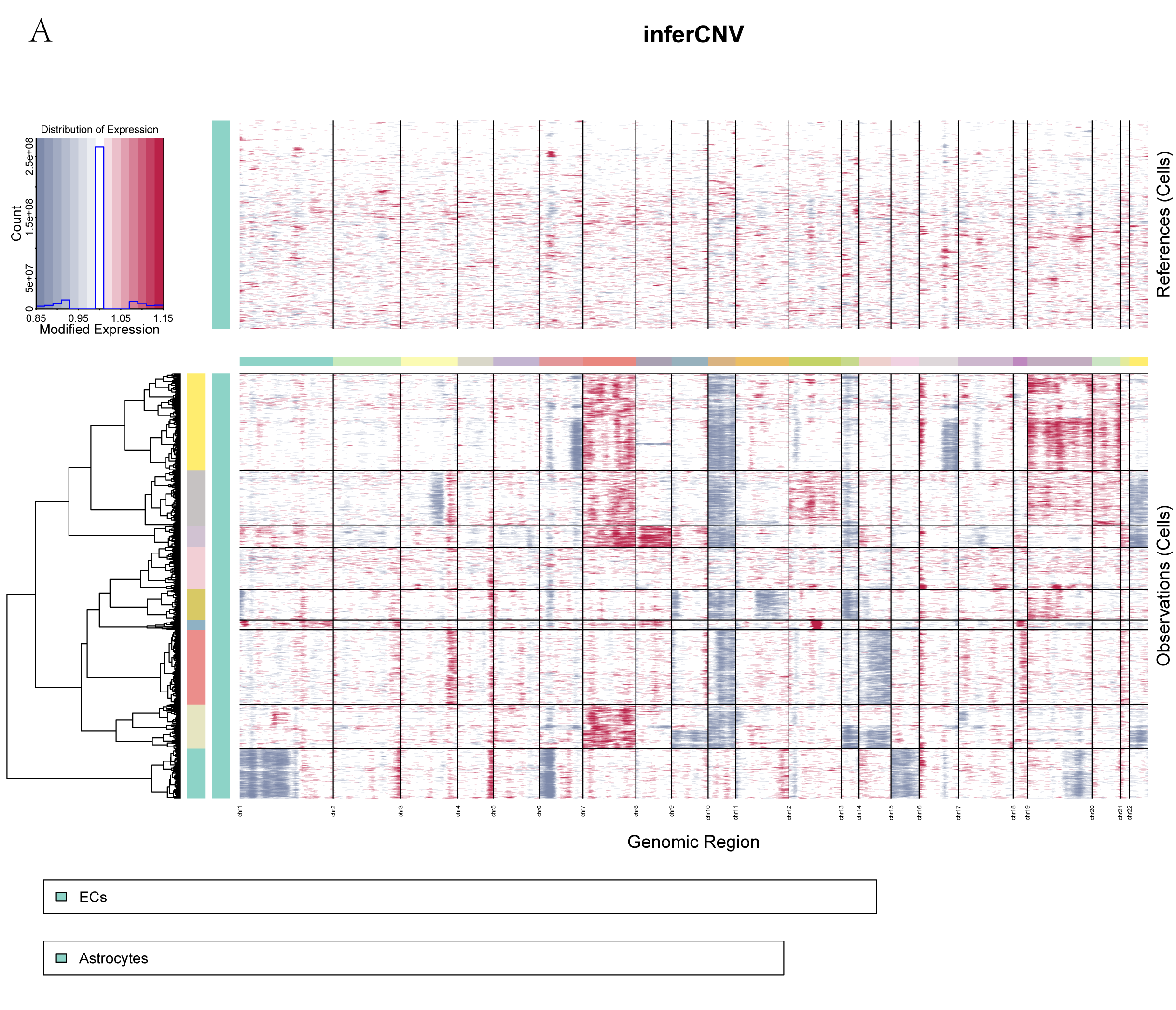

Supplement: Supplementary Figure 2 — Analysis of astrocytes through inferCNV. (A) The inferCNV analysis of astrocytes was visualized in a heatmap. Astrocytes with high levels of inferCNV were defined as astrocytoma. The red color represented high copy number variation (astrocytoma), while the blue color represented low copy number variation. [file Image2.tif]
